# A Meta-Analysis of the Relationship between *FGFR3* and *TP53* Mutations in Bladder Cancer

**DOI:** 10.1371/journal.pone.0048993

**Published:** 2012-12-13

**Authors:** Yann Neuzillet, Xavier Paoletti, Slah Ouerhani, Pierre Mongiat-Artus, Hany Soliman, Hugues de The, Mathilde Sibony, Yves Denoux, Vincent Molinie, Aurélie Herault, May-Linda Lepage, Pascale Maille, Audrey Renou, Dimitri Vordos, Claude-Clément Abbou, Ashraf Bakkar, Bernard Asselain, Nadia Kourda, Amel El Gaaied, Karen Leroy, Agnès Laplanche, Simone Benhamou, Thierry Lebret, Yves Allory, François Radvanyi

**Affiliations:** 1 Department of Urology, Hôpital Foch, Université de Versailles – Saint-Quentin-en-Yvelines, Suresnes, France; 2 Centre de Recherche, Institut Curie, Paris, France; 3 UMR144, CNRS, Paris, France; 4 Department of Biostatistics, Institut Curie, INSERM U900, Paris, France; 5 Unité 855, INSERM, Créteil, France; 6 Faculté des Sciences de Tunis, Tunis, Tunisia; 7 Institut Pasteur, Tunis, Tunisia; 8 Department of Urology, Hôpital Saint-Louis, APHP, Université Paris 7, Paris, France; 9 INSERM U944, Institut Universitaire d'Hématologie, Université Paris 7, Paris, France; 10 Department of Biochemistry, Hôpital Saint-Louis, APHP, Paris, France; 11 UMR7151, CNRS, Université Paris 7, Paris, France; 12 Department of Pathology, Hôpital Tenon, APHP, Paris, France; 13 Department of Pathology, Hôpital Foch, Suresnes, France; 14 Department of Pathology, Hôpital Saint-Joseph, Paris, France; 15 Department of Pathology, Hôpital Henri Mondor, APHP, Créteil, France; 16 Department of Urology, Hôpital Henri Mondor, APHP, Créteil, France; 17 Sinai University, Cairo, Egypt; 18 Hôpital Charles Nicole, Tunis, Tunisia; 19 INSERM U794/CNRS FRE2939, Institut Gustave Roussy, Villejuif, France; University of Colorado, United States of America

## Abstract

*TP53* and *FGFR3* mutations are the most common mutations in bladder cancers. *FGFR3* mutations are most frequent in low-grade low-stage tumours, whereas *TP53* mutations are most frequent in high-grade high-stage tumours. Several studies have reported *FGFR3* and *TP53* mutations to be mutually exclusive events, whereas others have reported them to be independent. We carried out a meta-analysis of published findings for *FGFR3* and *TP53* mutations in bladder cancer (535 tumours, 6 publications) and additional unpublished data for 382 tumours. *TP53* and *FGFR3* mutations were not independent events for all tumours considered together (OR = 0.25 [0.18–0.37], *p* = 0.0001) or for pT1 tumours alone (OR = 0.47 [0.28–0.79], *p* = 0.0009). However, if the analysis was restricted to pTa tumours or to muscle-invasive tumours alone, *FGFR3* and *TP53* mutations were independent events (OR = 0.56 [0.23–1.36] (*p* = 0.12) and OR = 0.99 [0.37–2.7] (*p* = 0.35), respectively). After stratification of the tumours by stage and grade, no dependence was detected in the five tumour groups considered (pTaG1 and pTaG2 together, pTaG3, pT1G2, pT1G3, pT2-4). These differences in findings can be attributed to the putative existence of two different pathways of tumour progression in bladder cancer: the *CIS* pathway, in which *FGFR3* mutations are rare, and the Ta pathway, in which *FGFR3* mutations are frequent. *TP53* mutations occur at the earliest stage of the *CIS* pathway, whereas they occur would much later in the Ta pathway, at the T1G3 or muscle-invasive stage.

## Introduction

Bladder cancer is one of the most common cancers worldwide. It is the fourth most prevalent cancer in men and the 11^th^ most prevalent cancer in women in the United States [1]. More than 90% of bladder cancers are carcinomas, which may present at different stages. Ta tumours are papillary, generally low-grade tumours, which do not invade beyond the basement membrane. Carcinoma *in situ* (CIS) is a flat tumour that does not invade the basement membrane but is always of high grade. T1 tumours invade the subepithelial connective tissue but do not infiltrate the underlying muscularis propria. T2, T3 and T4 tumours invade the muscularis propria, perivesical tissue and adjacent organs, respectively [2].

There is clinical and molecular evidence for the existence of two pathways of bladder tumour progression: the Ta and CIS pathways [3]–[6]. Ta tumours often recur after surgical resection, but they progress only rarely (5–10% of cases) and unpredictably to high-grade T1 tumours and then to muscle-invasive tumours. By contrast, CIS often progress (in about 50% of cases) to T1 and then to muscle-invasive tumours. About 80% of muscle-invasive tumours are thought to arise through the CIS pathway [5], [7]. Activating mutations of *FGFR3*, which encodes a growth factor receptor of the fibroblast growth factor receptor family, have been shown to be associated mostly with the Ta pathway of tumour progression, as such mutations have been reported in 65% of pTa tumours, less frequently in pT1 (33%) and pT2-4 tumours (22%) and not at all in CIS [4], [8], [9] [Table S1]. By contrast, *TP53* mutations are infrequent in Ta tumours (19% of cases) and frequent both in carcinoma *in situ* (52% of cases) and in muscle-invasive tumours (44% of cases) [3] [Table S2]. Conflicting results have been published concerning the relationship between *TP53* and *FGFR3* mutations. *TP53* and *FGFR3* mutations were initially thought to be essentially mutually exclusive, with *FGFR3* mutations specific to the Ta pathway and *TP53* mutations specific to the CIS pathway [10], [11]. However, Hernandez *et al.*, in a study of a large series of pT1G3 tumours (n = 119), which are particularly difficult to manage clinically, reported *FGFR3* and *TP53* mutations to be independently distributed [12]. This was interpreted as indicating that pT1 tumours constitute a particular group of bladder tumours, not all of which fit into the two known pathways of bladder tumour progression [6]. Several other studies have also investigated both *FGFR3* and *TP53* mutations and have reported the presence of both types of mutation in some tumours. The number of double mutants was small in each of these reports (5 in Zieger *et al.* [13]; 2 in Lindgren *et al.* [14], 5 in Lamy *et al.* [15]; 9 in Ouerhani *et al.* [16]). In all these studies, P53 mutations and FGFR3 mutations were found to be inversely associated with the grade and the stage of the tumour. Stage and grade can therefore act as potential confusion factors that may create spurious associations between the risks of each of mutations. Only large sample sizes with tumours of each grade and stage would allow for properly adjusting association analysis on these two factors.

We made use of all the previously published data (535 tumours) and unpublished data from the Henri Mondor, Foch, IGR, and Saint-Louis hospitals (382 tumours) for analyses of both *FGFR3* and *TP53* mutations, in a meta-analysis investigating the relationship between these two mutations. We investigated whether *FGFR3* and *TP53* mutations were dependent (*TP53* occurring more rarely in *FGFR3*-mutated tumours) or independent events (*TP53* occurring at similar frequencies in tumours with and without *FGFR3* mutations) in this large series of tumours. The frequency of *FGFR3* and *TP53* mutations depends strongly on tumour stage and grade. We therefore also performed the analysis on subgroups of tumours defined on the basis of stage, grade or both these parameters.

## Results

### Available data

We retained only tumours for which stage was documented from the various studies (published and unpublished) reporting mutations of both *FGFR3* and *TP53* in bladder cancer (Table 1). We excluded pure CIS and papilloma, as there were only two cases of CIS and one case of papilloma in total, in all the studies considered. We thus selected 917 tumours in total for study, and grade was documented for 827 of these tumours. Grades were provided in the study by Lamy *et al.*, but it was impossible to retrieve information about both stage and grade for a given tumour [15]. We therefore excluded the data from the study by Lamy *et al.* from the combined investigation of stage and grade. The stages and grades of tumours for each study are summarised in Tables S1 and S2 (published studies) and Table S3 (unpublished studies). In total, there were 350 pTa, 358 pT1, 209 pT2-4 and 88 G1, 249 G2 and 490 G3 tumours. For the combined analysis of stage and grade, we considered the following five categories of tumours: pTaG1 plus pTaG2 (as a single category), pTaG3, pT1G2, pT1G3 and pT2-4 tumours. We classified pTaG1 and pTaG2 tumours together, and pT2, pT3 and pT4 tumours together as, in each of these groups, the tumours concerned are considered to constitute the same clinical entity, regardless of grade.

**Table 1:** Summary of the materials and methods and patients sections of the various published and unpublished studies.

Study	Number of patients	Clinical characteristics	*FGFR3* analysis	*TP53* analysis	Pathological data
Mongiat-Artus UP	170	All cases from Ta to pT4 tumors	Allele-specific PCR* (Bakkar, 2005)	FASAY‡ (Ishioka, 1993, Flaman, 1995)	WHO grading
BladderCIT UP	222	Newly diagnosed cases (pTa, pT1); all cases (pT2 to pT4)	SNaPshot followed by sequencing** (van Oers, 2005)	Sequencing (exons 4 to 11) †	WHO grading Central review
Bakkar 2003	81	Newly diagnosed cases from pTa to pT4 tumors	DHPLC followed by sequencing (exons 7, 10, 15)***	DHPLC followed by sequencing (exons 2 to 11) ††	WHO grading Central review
Hernandez 2005	119	Newly diagnosed pT1G3 cases from a prospective study	Sequencing (exons 7, 10, 15)***	Sequencing (exons 4 to 9) †††	WHO grading Central review
Zieger 2005	85	All cases from pTa and pT1 tumors	Sequencing (exons 7, 10, 15)***	Sequencing (exons 5 to 8) ††††	Bergkvist classification
Lamy 2006	121	Newly diagnosed cases from pTa to pT4 tumors	Sequencing (exons 7, 10, 15)***	FASAY‡ (Ishioka, 1993, Flaman, 1995)	WHO grading
Lindgren 2006	75	All cases from pTa and pT1 tumors	RNA sequencing (exons 7, 10, 13, 15)***	RNA sequencing (exons 4 to 9) †††	WHO grading Central review
Ouerhani 2009	90	All cases from pTaG3 and pT1 to pT4 tumors	SNaPshot followed by sequencing** (van Oers, 2005)	Sequencing (exons 4 to 11) †	WHO grading Central review

UP indicates a study unpublished as of March 2012. Data for individual patients are available for the unpublished data and for Lindgren *et al.* paper (Table S4).

All cases: both newly diagnosed cases (incident cases) and cases of recurrence or progression were studied.

All *FGFR3* mutation analyses were performed on DNA, except for the study by Lindgren *et al.* (2006), in which mutations were assessed on RNA. For *TP53* mutation analysis, DNA was analysed, except for the study by Lindgren *et al.* (2006) and functional assays in yeast (FASAY), which were based on RNA (Ishioka *et al.* 1993).

‡ FASAY results were highly concordant with those for the sequencing of *TP53* (Camplejohn *et al.*, 2000).

* The FGFR3 mutations R248C, S249C, G372C, and Y375C studied account for 95% of all bladder tumours with *FGFR3* mutations.

** Mutations R248C, S249C, G372C, Y375C, A393E, K652E and K652Q, K652M, K652T account for 99.57% of all tumours with *FGFR3* mutations.

*** Mutations of exons 7, 10 and 15 of *FGFR3* account for 100% of all mutated tumors.

† Mutations of exons 4 to 11 of *TP53* account for 98% of all mutated tumors.

†† Mutations of exons 2 to 11 of *TP53* account for 100% of all mutated tumors.

††† Mutations of exons 4 to 9 of *TP53*account for 98% of all mutated tumors.

†††† Mutations of exons 5 to 8 of *TP53* account for 90% of all mutated tumors.

### Distribution of *FGFR3* and *TP53* mutations by stage and by grade

**Figure 1:**
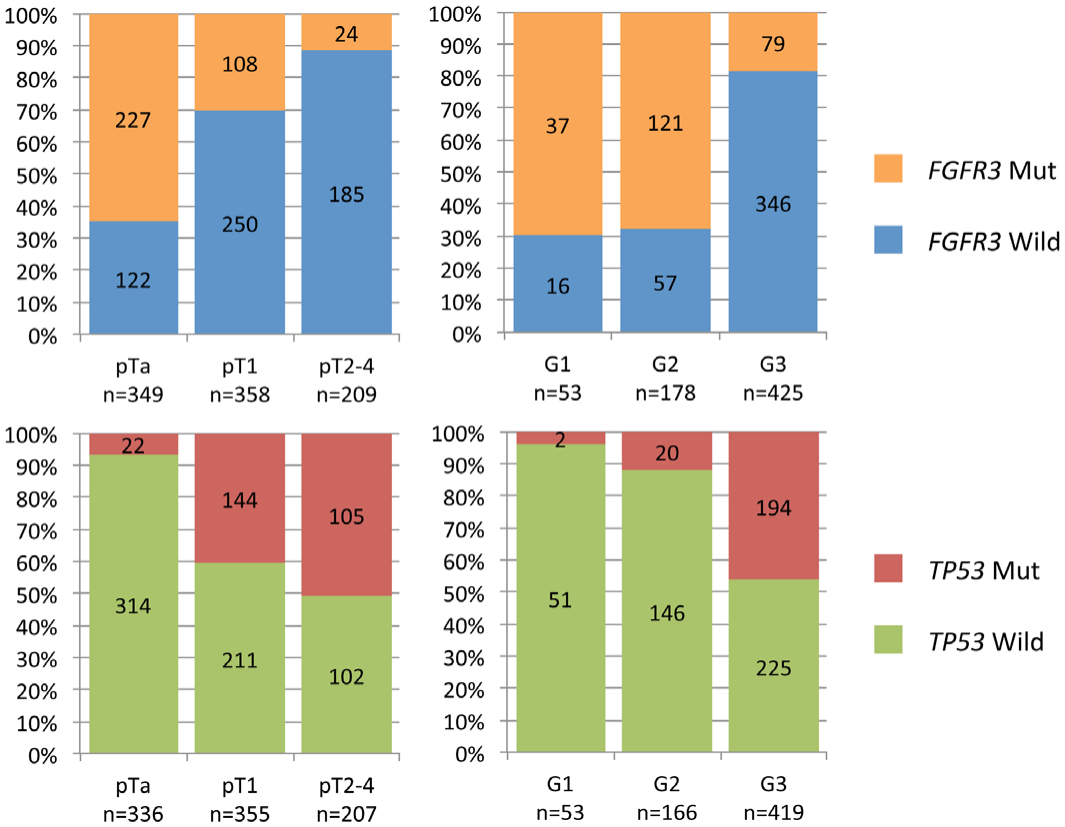
*FGFR3* and *TP53* mutation frequencies by stage (pT) or grade (G). Proportion of wild-type tumours and of tumours with *FGFR3* (upper row) and *TP53* (lower row) mutations as a function of pathological stage (left column) and grade (right column). The number of cases in each subgroup is indicated on the bars of the graph.

*FGFR3* mutation status was available for 916 of the 917 tumours with a documented stage and *TP53* mutation status was available for 898 of the 917 tumours. This meta-analysis, like many previous studies, showed an inverse relationship between *FGFR3* and *TP53* mutations for both stage and grade (Figure 1). The frequency of *FGFR3* mutations decreased with increasing stage and grade: 65% in pTa, 30.2% in pT1, 11.5% in pT2-4 and 69.8% in G1, a very similar rate in G2 (68%) and 18.6% in G3. These trends, for both stage and grade, were highly significant (*p*<0.0001 and *p*<0.0001, respectively). By contrast, the frequency of *TP53* mutations increased with increasing stage and grade: 6.55% in pTa, 40.6% in pT1, 50.7% in pT2-4, and 3.8% in G1, 12.05% in G2 and 46.3% in G3. These trends, for both stage and grade, were highly significant (*p*<0.0001 and *p*<0.0001 respectively), suggesting that stage and grade may be confounding factors.

### Association between *FGFR3* and *TP53* mutations, adjusting for stage or for grade

**Figure 2:**
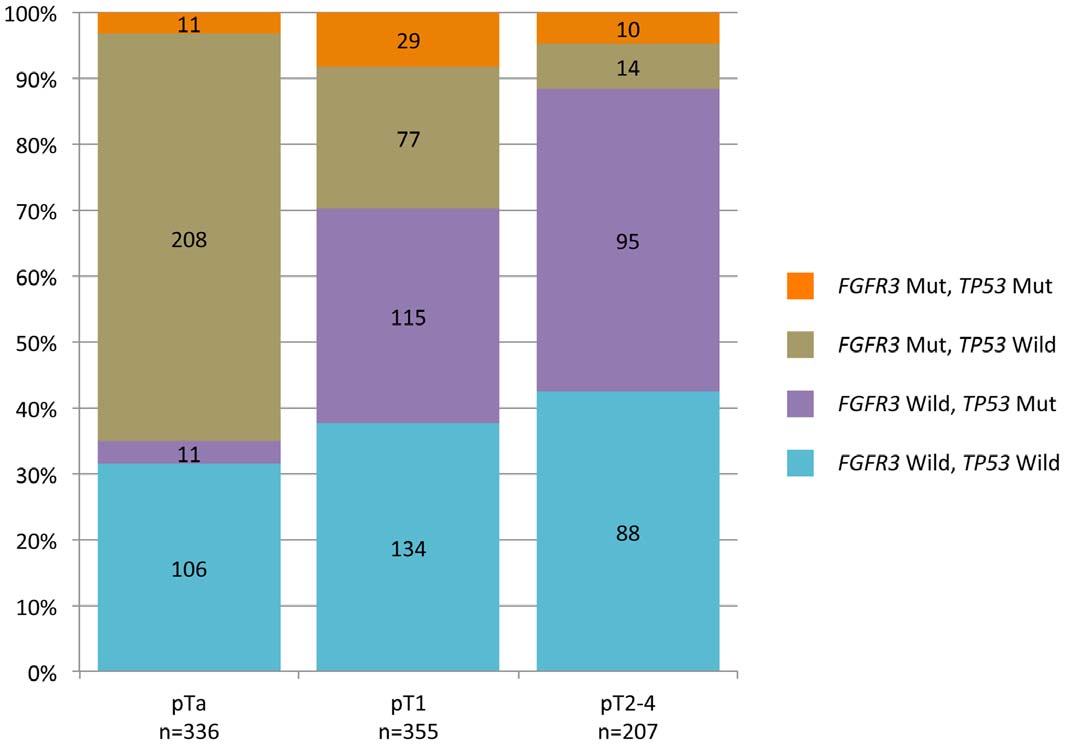
Combined *FGFR3* and *TP53* mutation frequencies by stage (pT). Proportion of tumours with both *FGFR3* and *TP53* mutations (orange), with mutated *FGFR3* and wild-type *TP53* (grey), with wild-type *FGFR3* and mutated *TP53* (purple), or with wild-type *FGFR3* and wild-type *TP53* (blue), as a function of pathological stage. The number of cases in each subgroup is indicated in the corresponding rectangle.

We then studied mutation status for both *FGFR3* and *TP53*, as a function of stage (Figure 2). For pTa tumours, the most common of the four possible groups (wild-type *FGFR3* plus wild-type *TP53*, wild-type *FGFR3* plus mutated *TP53*, mutated *FGFR3* plus wild-type *TP53*, mutated *FGFR3* plus mutated *TP53*) was tumours with mutated *FGFR3* and wild-type *TP53* (208/336; 61.9% of cases), followed by tumours wild-type for both *FGFR3* and *TP53* (106/336; 31.5% of cases). A small number of tumours had *TP53* mutations and were either wild-type for *FGFR3* (11/336; 3.3%) or mutated for *FGFR3* (11/336; 3.3%). For pT1 tumours, the two most common groups were tumours wild-type for both *FGFR3* and *TP53* (134/355; 37.7% of cases) or wild-type for *FGFR3* and mutated for *TP53* (115/355; 32.4% of cases). For invasive tumours (pT2-4), the two most common groups were also tumours wild-type for both *FGFR3* and *TP53* (88/207; 42.5% of cases) or wild-type for *FGFR3* and mutated for *TP53* (95/207; 45.9% of cases).

We then investigated whether *FGFR3* and *TP53* mutations were independent events. We defined four groups (wild-type *FGFR3* plus wild-type *TP53*, wild-type *FGFR3* plus mutated *TP53*, mutated *FGFR3* plus wild-type *TP53*, mutated *FGFR3* plus mutated *TP53*). We carried out a Mantel-Haenszel test, stratified for stage, to determine whether the proportion of tumours with *TP53* mutations differed significantly between tumours with wild-type *FGFR3* and tumours with *FGFR3* mutations, assuming a common association for each stage. For all tumours considered together, we found a strong association between *FGFR3* and *TP53* mutations (OR = 0.49 [0.33, 0.72], *p* = 0.001), such that the odds of a given *FGFR3*-mutated tumour having a *TP53* mutation were half those for *FGFR3* wild-type tumours. The Briant and Day interaction test suggested that the association did not differ significantly across strata (*p* = 0.72). In subgroup analysis, *FGFR3* and *TP53* mutations were not significantly associated in pTa tumours (*p* = 0.20) or in pT2-4 tumours (*p* = 0.345), but these two types of mutation were strongly associated, even after Bonferroni correction (significance level for the three tests  =  0.017) in pT1 tumours (ORa = 0.52 [0.30, 0.88], *p* = 0.0009) (Table 2).

**Table 2:** Association between *FGFR3* and *TP53* mutations according to stage and grade.

*Stage pT*	ORa	95% Wald Confidence Limits		Fisher's exact test *P*-value
**pTa** (*n* = 336)	0.56	0.23	1.36	0.20
**pT1** (*n* = 355)	0.52	0.30	0.88	<0.01
**pT2-4** (*n* = 207)	0.66	0.28	1.67	0.34
***Grade G***				
**G1**	0.41	0.03	6.81	0.51
**G2**	0.58	0.26	1.3	0.19
**G3**	0.58	0.35	0.9	0.02

ORa = odds ratio estimate.

It was possible to carry out a similar analysis for grade, as opposed to stage, for seven studies (Mongiat-Artus UP, BladderCIT UP, Bakkar *et al.*, 2003, Hernandez *et al.*, 2005, Lamy *et al.*, 2006, Lindgren *et al.*, 2006, Ouerhani *et al.*, 2009) including 638 patients for whom mutation status data were available for both *TP53* and *FGFR3*. Some heterogeneity in the association was detected between grades (*p* = 0.05 in the Briant and Day test). We found an association between *FGFR3* and *TP53* mutations only in G3 tumours (OR = 0.57 [0.35–0.93], *p* = 0.0245), but this finding was of borderline difference after adjusting for multiple testing.

### Association between *FGFR3* and *TP53* mutations, adjusting for combined categories of both stage and grade

**Figure 3:**
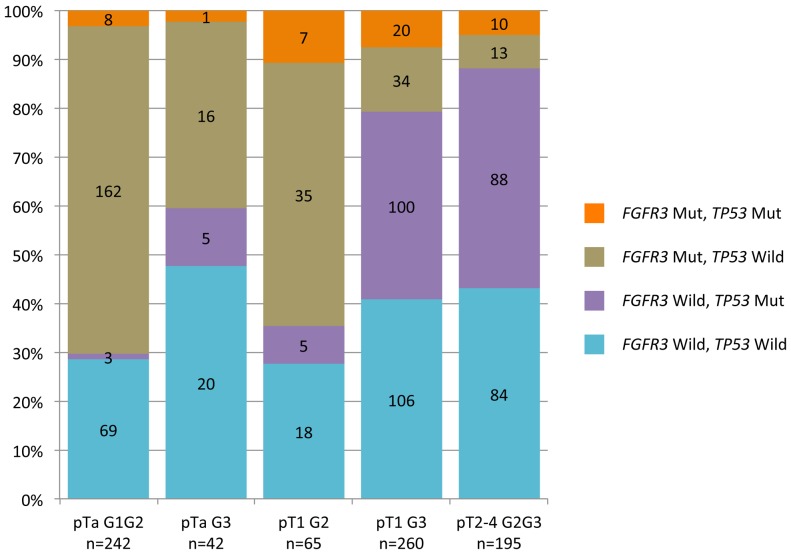
Combined *FGFR3* and *TP53* mutation frequencies according to the stage/grade group. Proportion of tumours with both *FGFR3* and *TP53* mutations (orange), with *FGFR3* mutations and wild-type *TP53 wild-type* (grey), with wild-type *FGFR3* and mutated *TP53* (purple), or with wild-type *FGFR3* and wild-type *TP53* (blue), as a function of stage/grade. The number of cases in each subgroup is indicated in the corresponding rectangle.

We then assigned the tumours to groups on the basis of both stage and grade (Figure 3). We defined five categories: pTaG1 and pTaG2 (a single category), pTaG3, pT1G2, pT1G3, pT2-4 (all stages, a single category). This division is based on that used in clinical practice. There was a strong association between *TP53* mutations and category, as the frequency of *TP53* mutations was 4.55% in pTaG1-2, 14.3% in pTaG3, 18.5% in pT1G2, 46.15% in pT1G3 and 50.25% in pT2-4 tumours, the largest difference thus being that between pT1G2 and pT1G3 tumours. An association between *FGFR3* mutation status and category was also observed, although the trend was less clear-cut, because of the strong influence of both grade and stage on FGFR3 mutation rate: the frequency of *FGFR3* mutation was very similar in pTaG1-2 tumours (70.2%) and pT1G2 tumours (64.6%), but significantly lower in pTaG3 tumours (40.5%) (χ^2^ test; *p* = 0.0001). The frequency of *FGFR3* mutation was even lower in pT1G3 tumours (20.8%) and pT2-4 tumours (11.8%).

We then studied the association between *FGFR3* and *TP53* mutations in the five tumour stage/grade categories (table 3). As before, we defined four groups within each category (wild-type *FGFR3* plus wild-type *TP53*, wild-type *FGFR3* plus mutated *TP53*, mutated *FGFR3* plus wild-type *TP53*, mutated *FGFR3* plus mutated *TP53*). Overall, no significant association was detected after adjusting for stage and group (OR = 0.69; 95% CI = 0.44 to 1.08, Mantel-Haenszel test = 0.075) and we detected no statistically significant heterogeneity across strata (*p* = 0.41 for heterogeneity). *FGFR3* and *TP53* mutations were considered to be independent events in all categories (Table 3), as the proportion of tumours with mutated *TP53* did not differ significantly between the tumours with wild-type and mutated *FGFR3* (Table 4).

**Table 3:** Association between *FGFR3* and *TP53* mutations according to the stage/grade group.

	Odds ratio estimates			
*Group TxGy*	OR	95% Wald Confidence Limits		Fisher's exact test *P*-value
**pTaG1G2** (*n* = 242)	1.16	0.3	4.6	0.85
**pTaG3** (*n* = 42)	0.47	0.01	5.4	0.37
**pT1G2** (*n* = 65)	0.55	0.14	2.11	0.74
**pT1G3** (*n* = 260)	0.65	0.34	1.24	0.17
**pT2-4G2G3** (*n* = 195)	1.25	0.44	1.25	0.83

**Table 4:** *TP53* mutation rates in *FGFR3*-wild-type and FGFR3-mutated tumours, according to a combination of stage and grade.

	*TP53* mutation rate				
	pTaG1G2 (*n* = 242)	pTaG3 (*n* = 42)	pT1G2 (*n* = 65)	pT1G3 (*n* = 260)	pT2-4 (*n* = 195)
*FGFR3* mutant	4.7% (8/170)	5.9% (1/17)	16.7% (7/42)	37% (20/54)	43.5% (10/23)
*FGFR3* wild-type	4.2% (3/72)	20% (5/25)	21.7% (5/23)	48.5% (100/206)	51.2% (88/172)
*P*-value Fisher's exact test	0.99	0.37	0.74	0.17	0.51

We assessed the robustness of the results, by carrying out the same analysis while accounting for the potential study effect. Similar results were obtained despite the continuity correction required to correct for too small sample sizes in some defined by the combination of stage/grade.

## Discussion

Due to their inverse distributions as a function of stage and grade and the small number of double-mutated tumours (*FGFR3* mutated, *TP53* mutated) observed in small series, *FGFR3* and TP53 mutations had been reported to be mutually exclusive events, with *FGFR3* mutation strongly associated with the Ta pathway and *TP53* mutation strongly associated with the CIS pathway [10], [11]. The group of Real showed for the first time, in a study of a large series of tumours, that *FGFR3* and *TP53* mutations were independent events in pT1G3 tumours (*n* = 119) [12]. Our meta-analysis of 917 tumours (including all published data (535 tumours) plus an additional series of 382 tumours of all stages and grades) confirms and extends the findings of Hernandez *et al.*, by showing that *FGFR3* mutations and *TP53* mutations are independent events not only in pT1G3 tumours (confirmed here for 260 pT1G3 tumours) but also in pTa, pT1G2 and muscle-invasive tumours (pT≥2). By contrast, *FGFR3* mutations and *TP53* mutations were not independent events if we considered all tumours together, without accounting for stage and grade (*p* = 0.0001), or all pT1 tumours together (pT1G2 and pT1G3 tumours) (*p* = 0.0009).

The two known pathways of tumour progression and the different frequencies of *FGFR3* and *TP53* mutations in the different groups of tumours defined on the basis of grade and stage may account for these observations. At an early stage (pTaG1 and pTaG2), the frequency of *FGFR3* mutation was high in the Ta low-grade pathway, whereas that of *TP53* mutation was very low. If these tumours progress to muscle-invasive tumours, they will carry *TP53* mutations at a frequency similar to that in tumours of the CIS pathway. Indeed, in our meta-analysis, muscle-invasive *TP53* mutations were found in 42% of tumours with *FGFR3* mutation (10/24). This proportion is lower than the 52% (95/183) of muscle-invasive tumours with wild-type *FGFR3* observed, but not significantly so. For tumours of the Ta pathway, the frequency of *TP53* mutations seems to increase gradually with stage, from pTa (11/219; 5% of tumours with *FGFR3* mutations also have *TP53* mutations) to pT1 (29/106; *TP53* mutations in 27% of tumours) and pT2-4 tumours (10/24; *TP53* mutations in 42% of tumours). Grade also seems to be important, as, for *FGFR3*-mutated tumours, the frequency of *TP53* mutation was 17% in pT1G2 tumours and 37% in pT1G3 tumours. The frequency of *FGFR3* mutation was found to be very similar in pT1G2 and pTa G1G2 tumours, suggesting that most pT1G2 tumours are derived from the Ta pathway rather than the CIS pathway. Consistent with this hypothesis, the frequency of *TP53* mutation in pT1G2 tumours, although higher than that in pTa tumours (18% versus 7%), was found to be much lower than that reported in cases of dysplasia or CIS tumours (65–72%) [3], [17]. Thus, the lack of independence of *FGFR3* and *TP53* mutations when all pT1 tumours are considered together may reflect the two different pathways giving rise to tumours of this stage, with different frequencies of *TP53* mutation.

There are also more complex explanations for the findings of this meta-analysis. Mutation data for a series of tumours provide only a snapshot of the situation, from which the progression of individual tumours is inferred. Definitive validation of the model proposed will require extensive studies of *TP53* and *FGFR3* mutations in multiple tumours from the individual patients, including both non-muscle invasive and muscle-invasive tumours.

It should be noted that a high proportion of bladder tumors present a wild-type phenotype (no *FGFR3* or *TP53* mutations): 32% of Ta tumors, 40% of T1 tumors and 43% of T2-4 tumors). These tumours probably have mutations in genes other than *FGFR3* or *TP53*, but with a similar effect (activation of the *FGFR3* signaling pathway in Ta tumors of low grade and mutations of genes causing genetic instability in high-grade or high-stage tumors). *RAS* mutations, which are observed in about 10% of bladder cancers and are never found with *FGFR3* mutations, are thought to affect the FGFR3 signaling pathway [5], [18]. *MDM2* amplifications, which occur in about 6% of T1 and muscle-invasive bladder cancers [19], should lead to *TP53* inactivation. Other mutations recently identified [20], or yet to be identified may also account for the absence of *FGFR3* and *TP53* mutations.

We recently showed that most (80%) muscle-invasive tumours with *FGFR3* mutations harbour homozygous *CDKN2A* deletions, resulting in the loss of both P16INk4A and P14ARF [21]. The loss of P14ARF should lead to MDM2 activation, thereby decreasing TP53 levels. However, in tumours with *FGFR3* mutations, homozygous *CDKN2A* deletion and *TP53* mutation may occur together (data not shown), suggesting that these two events are not alternative mechanisms of inactivation for the same pathway. Indeed, the loss of *CDKN2A* also leads to the deletion of *p16INk4A*, which encodes a cyclin kinase inhibitor controlling RB protein activity through CDK4 in G1/S. Furthermore, p14ARF may have several TP53-independent activities [22] and *TP53* mutations may have gain-of-function effects [23]. Thus, the various events (*CDKN2A* loss and *TP53* mutation) may contribute in an additional manner to the transformed phenotype.

Meta-analyses are often performed on clinical data, but more rarely on biological data. Meta-analysis is much more powerful than individual analysis but has several drawbacks: 1) Different studies use different methodologies. Indeed, in the studies analysed here, different methods were used to assess the frequency of both *FGFR3* (snapshot or sequencing) and *TP53* (direct sequencing or FASAY) mutations. Furthermore, different exons were explored in different studies: exons 7, 10, 15 or 7, 10, 13, 15 for *FGFR3* and exons 5 to 8, 4 to 9, 4 to 11 or 2 to 11 for *TP53*. Differences due to the choice of exons studied should be negligible; mutations of exon 15 of *FGFR3* account for only 3% of all identified mutations when exons 7, 10, and 15 are considered, and mutations in exons 2, 3, 4, 9, 10 and 11 of *TP53* mutations account for only 6.3% of all identified mutations when exons 2 to 11 of this gene are considered. FASAY and direct sequencing are very different in nature (FASAY being a functional assay and more sensitive), but they give very similar results [24]. Most of the tumours explored by the FASAY method in our analysis were pTaG1-G3 or pT1G2 tumours. A comparison of the FASAY data with direct sequencing data for the same tumours showed the frequencies of *TP53* mutation obtained with these two techniques to be very similar. 2) This meta-analysis may also have been biased by the lack of review of stage and grade determination. The stages and grades assigned to tumours may therefore differ between the studies included in the meta-analysis. This could be problematic for pT1 tumours, because some pTa tumours may have been overstaged and classified as pT1 tumours [25].

This meta-analysis provides a good example of the effect of confounding factors that are not taken into account. Such factors may not only modify the magnitude of any association, but also create spurious associations. Larger sample sizes and more detailed data are therefore required for a valid statistical analysis. In such conditions, meta-analysis is an effective approach, making it possible to draw reliable conclusions, because it builds on the strengths of several studies that may not be sufficiently powerful individually to generate conclusive results. However, meta-analyses can only take into account the confounding factors that were actually measured. Other unknown variables may affect the relationship between the pathway and disease severity and remain undetected. All the publications retained for this analysis had a high quality of reporting, making it possible to extract the relevant information.

Despite the known weaknesses of meta-analysis, the large sample and the homogeneity of the results after adjustment add weight to our conclusions: we found that *FGFR3* and *TP53* mutations occurred independently when stage and grade were taken into account, and that the frequency of *TP53* mutation was high in pT1G3 and pT2-4 tumours, regardless of the presence or absence of *FGFR3* mutations in these tumours. Thus, *TP53* mutations can occur in Ta pathway tumours, when these tumours progress. However, the time frame of *TP53* mutation differs considerably between the two pathways: *TP53* mutations occur before basement membrane invasion in the carcinoma *in situ* pathway, whereas they probably occur after or during basement membrane invasion in the Ta pathway. Our findings also indicate that care is required in the analysis of bladder tumours, as both pathways of tumour progression for bladder cancers must be taken into account when interpreting data.

## Materials and Methods

### Studies

Studies of adult patients with bladder carcinoma were eligible for inclusion in the analysis if they reported *FGFR3* and *TP53* mutations as a function of stage. Paediatric patients (13 urothelial carcinoma cases) were excluded because the process of carcinogenesis in these patients is probably different from that in adult cases [26].

**Figure 4:**
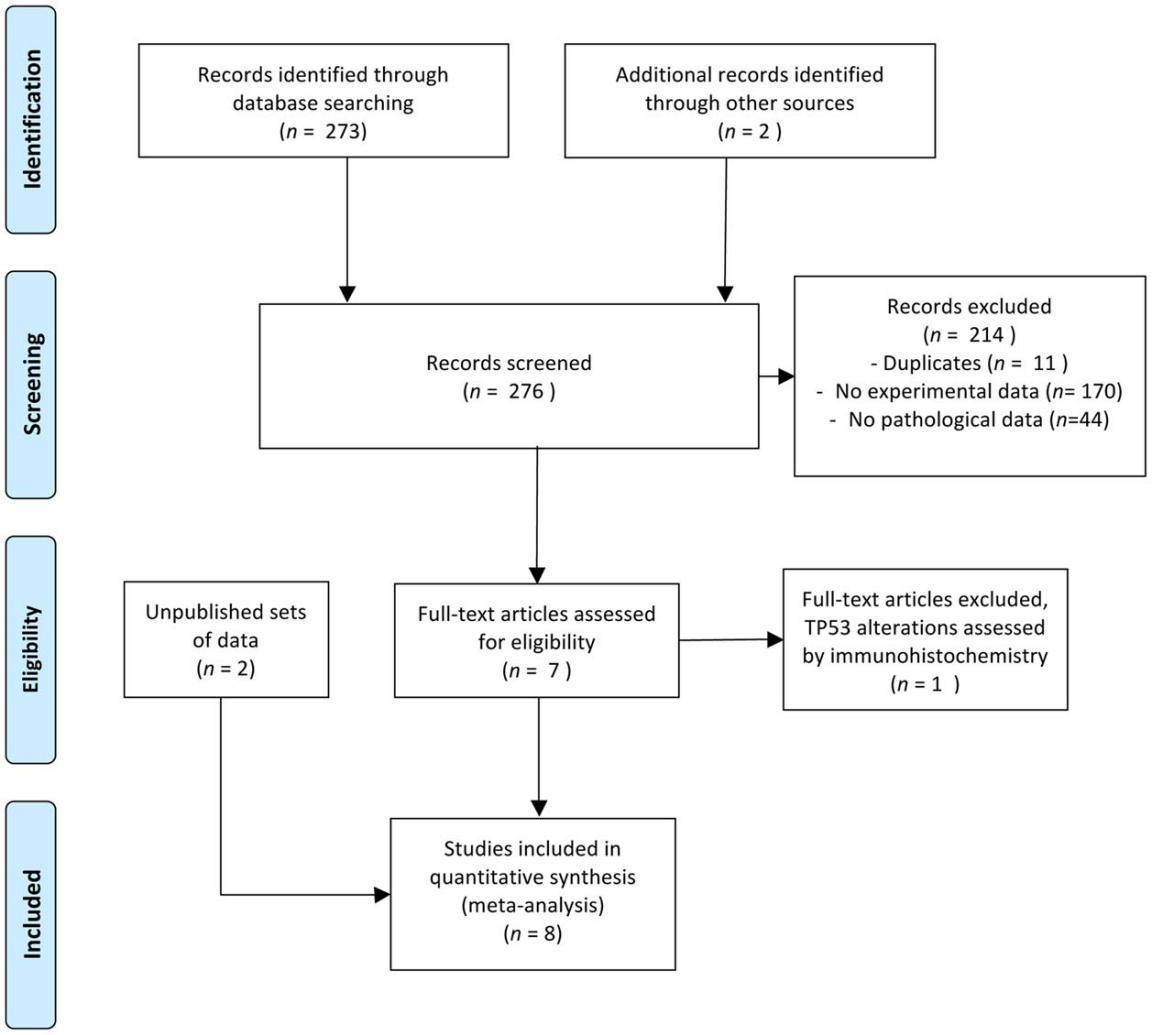
PRISMA 2009 flow diagram of the study selection process.

We searched databases (MEDLINE, PubMed, Embase) with cross-referenced search terms customised for each of the search engines used, to identify relevant articles published in English and dealing with *FGFR3* and *TP53* mutations in bladder carcinomas. We searched the reference lists of review articles manually to identify additional publications. In accordance with PRISMA guidelines [27], the eight identified studies including a total of 963 patients are presented in Figure 4. All but one (Hernandez *et al.*, 2005) of these studies were retrospective single- or multicenter studies investigating the association between gene mutations and pathways in urothelial cell carcinoma. One study focused on the pT1G3 stage (Hernandez *et al.*, 2005), whereas the others included patients at early stages (pTa and pT1) (Lindgren *et al.* 2006), late stages (pT1-4) (Ouerhani *et al.*, 2009) or at any stage. Three studies concerned newly diagnosed patients (Bakkar 2003, Hernandez *et al.*, 2005; Lamy 2006). Two of these studies have not yet been published and are denoted below by the name of the main investigator or the program supporting the study plus “UP” (for unpublished; thus, Mongiat-Artus UP and BladderCIT UP).

Information about treatment (irradiation or chemotherapy) before biopsy was unavailable for a large proportion of the patients. We therefore included all patients, regardless of their prior treatment. The main characteristics of these eight studies are summarised in Table 1. We refer to the publications for all details in the cases of published data. In the Bladder CIT UP study, the pTa and pT1 tumours were from incident cases and the pT2-4 tumours were from both newly diagnosed and progressing cases (patients with a history of previous non-muscle-invasive tumours). *FGFR3* mutations were assessed by the SNaPshot technique in the BladderCIT UP study [28] and by allele-specific PCR [29] in the Mongiat-Artus UP study. *TP53* mutations were assessed by direct sequencing on both strands, followed by confirmation of the identified mutations in an independent PCR in the BladderCIT UP study and by the FASAY method [30], [31] followed by confirmation of the identified mutations by an independent PCR in the Mongiat-Artus UP study.

We did not include the study by van Rhijn *et al.* (2004) [32] because TP53 alterations were assessed by immunohistochemistry in this study and it has been shown that there is only 57 to 71% similarity between the alterations detected by mutation assessment and those detected by immunohistochemistry [Table S3].

The following variables were collected: *TP53* and *FGFR3* mutations, stage and grade of the disease and the type of mutation. Data for individual patients were available for four studies (Mongiat-Artus UP, BladderCIT UP, Lindgren *et al.*, 2006, Ouerhani *et al.*, 2009) [Table S4 and S5]. For the other reports, data were extracted directly from the publications by cross-referencing tables. For three studies (Bakkar *et al.*, 2003; Lindgren *et al.*, 2008; Ouerhani *et al.*, 2009), the authors were contacted and provided additional information.

### Biological and pathological data

The frequencies of *FGFR3* and *TP53* mutations by grade and tumour stage were recorded in all studies. Data were extracted from the publications or from the raw data, when available [Table S4 and S5].

Grading was assessed with the WHO 1973 classification in all but one of the studies (Zieger *et al.*, 2005), the remaining study using the Bergkvist classification. Grades 1 and 2 of this classification were classified as grades 1 and 2, respectively, of the WHO 1973 grading. Grades 3 and 4 (one tumour) of the Bergkvist classification were considered to correspond to grade 3 of the WHO 1973 grading. In five studies, a central review by a senior pathologist was reported in the publication or in the analysis report for unpublished data. No such information was available for the other three studies.

In cases in which multiple tumour samples from individual patients were studied (BladderCIT UP, Zieger *et al.*, 2005), the first tumour diagnosed was retained for the analysis. In the Zieger series, only two tumours were reported to have different mutations in the first and second sample: one tumour had *FGFR3* mutations in the second biopsy sample but not the first, whereas the other had TP53 mutations only in the last sample. A sensitivity analysis, based on the use of the last sample rather than the first one, was carried out and gave very similar results.

### Statistical analysis

Tumours were classified into three different groups for stage (T): Ta, T1 and T2-4. CIS and papilloma were excluded because too few cases were reported for reliable analysis. We also established five groups for exploration of the combination of stage and grade (G). These “TG” groups — TaG1-2, TaG3, T1G2, T1G3 and T2-4 — are clinically relevant. Most TaG1 and TaG2 tumours are treated by simple resection. T2-4 tumours are treated by cystectomy. T1G3 tumours are the most aggressive of the non-muscle-invasive tumours and TaG3 tumours behave very differently from TaG1 and TaG2 tumours [33], [34].

We analyzed the relationships of *FGFR3* or *TP53* mutation frequency with stage and grade. We then focused on the association of mutations of *FGFR3* and *TP53*.

We initially analysed the association of mutation rates for these two genes stratified on stage and grade separately.

Overall associations were tested in adjusted Mantel-Haenszel tests, after checking for interactions of associations by clinical T stage or by grade. Interactions were explored using Cochran homogeneity tests. In cases of interaction, if association was estimated to be in opposite direction, subgroup analysis by stratum was performed. Fisher's exact tests were used when the sample size per stratum was too small.

The magnitude of the association is expressed as an adjusted odds ratio (OR), comparing the odds of *FGFR3* mutation in the tumours with wild-type and mutated *TP53*. Adjusted ORs were estimated from the contingency table.

A significance threshold of 5% was used for all global tests. Subgroup analyses (defined by stage, grade or a combination of both) were adjusted for multiple testing, by the Bonferroni method, assuming the tests to be independent.

## Supporting Information

Table S1 Overview of FGFR3 mutations studies in bladder carcinoma.
(DOC)

Table S2 Overview of TP53 mutations studies in bladder carcinoma.
(DOC)

Table S3 Overview of FGFR3 and TP53 mutations in bladder carcinoma in the two unpublished studies.
(DOC)

Table S4 Available individual data from unpublished, Bakkar, Lindgren, Ouerhani, and Zieger studies.
(DOC)

Table S5 Joint distribution of FGFR3 and P53 mutations frequencies by stage (T) and grade (G) group.
(DOC)
