# Supplementary material for: A Meta-Analysis of the Relationship between FGFR3 and TP53 Mutations in Bladder Cancer
Source: PLoS One. 2012 Dec 13;7(12):e48993. doi: 10.1371/journal.pone.0048993 (PMC3521761; doi:10.1371/journal.pone.0048993)
Supplement: Table S1 — Overview of FGFR3 mutations studies in bladder carcinoma. (DOC) [file pone.0048993.s001.doc]

**Supplementary Table 1: Overview of *FGFR3* mutations studies in bladder carcinoma**

| Ref. PMID | Techniques | Exon | Pathological stages and grades | Frequencies of mutations (%) | Frequencies of mutations by Exon (%) |
| --- | --- | --- | --- | --- | --- |
| 16061860  Hernandez, 2005 | Sequencing | 7, 10, 15 | 119 pTaG3 | **20/119 (16.8%)** | Exon 7: 17 (85%)  Exon 10: 3 (15%)  Exon 15: 0 (0%) |
| 16278391  Zieger, 2005 | Sequencing | 7, 10, 15 | pTaG1-2 = 29  pT1G3 or CIS = 23  Recurrent (Rc) pT1G3 or CIS = 22  pT≥2 = 41 | **55/115 (47.8%)**  pTaG1-2 = 20/29 (69.0%)  pT1G3/CIS = 12/23 (52.2%)  Rc pT1G3/CIS = 9/22 (40.9%)  pT≥2 = 13/41 (31.7%) | Exon 7: 29 (52%)  Exon 10: 26 (48%)  Exon 15: 0 (0%) |
| 11395371  Billerey, 2001 | SSCP and sequencing | 7, 10, 15, 19 | pTa = 50, pT1 = 19, pT≥2 = 43, pTis = 20  Gr. 1 = 32, Gr. 2 = 29, Gr. 3 = 71 | **48/132 (36.4%)**  pTaG1 = 27/32 (84%)  pTaG2 = 9/13 (69%)  pTaG3 = 1/5 (20%)  pT1G1 = 0/0 (0%)  pT1G2 = 2/3 (67%)  pT1G3 = 2/16 (12.5%)  pT≥2G2 = 5/13 (38%)  pT≥2G3 = 2/30 (7%) | Exon 7: 41 (85.4%)  Exon 10: 5 (10.4%)  Exon 15: 2 (4.2%) |
| 12237885  van Rhijn, 2002 | SSCP and sequencing | 7, 10, 15 | LMP = 12, pTaG1 = 79 | **77/91 (84.6%)**  LMP = 9/12 (75%)  pTaG1 = 68/79 (86.1%) | Exon 7: 56 (72.7%)  Exon 10: 19 (24.7%)  Exon 15: 2 (2.6%) |
| 11314002  Sibley, 2001 | SSCP and sequencing | 7, 10, 15 | 63 tumours and 18 cell lines | **30/81 (37%)**  Tumours = 26/63 (41%)  Cell lines = 4/18 (22%) | Exon 7: 23 (76.7%)  Exon 10: 4 (13.3%)  Exon 15: 3 (10.0%) |
| 11245416  van Rhijn, 2001 | SSCP and sequencing | 7, 10, 15 | pTa = 53, pT1 = 4, pT≥2 = 15 | **34/72 (47.2%)**  pTa = 34/53 (64.2%)  pT1 = 0/4  pT≥2 = 0/15 | - NR - |
| 15026322  van Rhijn, 2004 | SSCP and sequencing | 7, 10, 15, 19 | pTa = 171, pT1 = 55, pT≥2 = 34 | **153/260 (59%)**  pTa = 131/171 (77%)  pT1 = 17/55 (31%)  pT≥2 = 5/34 (15%) | Exon 7: 117 (76.5%)  Exon 10: 32 (20.9%)  Exon 15: 4 (2.6%)  Exon 19: 0 (0%) |
| 15897885  Jebar, 2005 | SSCP and sequencing | 7, 10, 15 | pTa = 39, pT1 = 23, pT≥2 = 23, pTis = 1  unknown = 12  Gr. 1 = 45, Gr. 2 = 31, Gr. 3 = 18  unknown = 4 | **54/98 (55.1%)** | Exon 7: 43 (79.6%)  Exon 10: 10 (18.5%)  Exon 15: 1 (1.9%) |
| 16532037  Lindgren, 2006 | SSCP and sequencing | 7, 10, 13, 15 | TaG1 = 25, TaG2 = 29, TaG3 = 3,  T1G2 = 10, T1G3 = 8 | **46/75 (61.3%)**  TaG1 = 20/25 (80%)  TaG2 = 18/29 (62%)  TaG3 = 0/3 (0%)  T1G2 = 7/10 (70%)  T1G3 = 1/8 (12%) | - NR - |
| 10471491  Cappellen, 1999 | SSCP and sequencing | All | - NR - | **9/76 (12%)**  TaG2 = 1/?  T1aG1 = 2/?  T1aG3 = 1/?  T1bG3 = 1/?  T2G3 = 1/?  T3G2 = 2/? | Exon 7: 6 (67%)  Exon 10: 2 (22%)  Exon 15: 1 (11%) |
| 14678961  Bakkar, 2003 | DHPLC and sequencing | 7, 10, 15 | pTa = 31, CIS = 1, pT1 = 30, pT2-4 = 19  G1 = 10, G2 = 29, G3 = 42 | **32/81 (40%)** | Exon 7: 22 (69%)  Exon 10: 7 (22%)  Exon 15: 3 (9%) |
| 17085196  Lamy, 2006 | Sequencing | 7, 10, 15 | Ta = 47, T1 = 35, T2 = 25  Gr. 1 = 13, Gr. 2 = 28, Gr. 3 = 66 | **43/107 (40%)**  Ta = 31 (66%)  T1 = 9 (26%)  T2 = 3 (12%)  Gr. 1 = 7 (54%)  Gr. 2 = 23 (85%)  Gr. 3 = 13 (20%) | Exon 7: 29 (67%)  Exon 10: 11 (26%)  Exon 15: 3 (7%) |
| 19156776  Kompier, 2009 | SNaPshot assay |  | pTa = 74, pT1 = 36, CIS = 5  Gr. 1 = 45, Gr. 2 = 43, Gr. 3 = 29 | **73/118 (62%)**  Ta = 59 (80%)  T1 = 10 (28%)  CIS = 4 (80%)  Gr. 1 = 38 (84%)  Gr. 2 = 28 (65%)  Gr. 3 = 7 (24%) | - NR - |
| 19722178  Bakkar, 2010 | SNaPshot assay |  | PTa = 75, pT1 = 52, pT2-4 = 43  LMP = 6, Low Gr. = 41, High Gr. = 141  Patients with exposure to polycyclic aromatic hydrocarbons | **66/170 (39%**)  pTa = 53 (71%)  pT1 = 10 (19%)  pT2-4 = 3 (7%)  LMP + Low Gr. = 36 (77%)  High Gr. = 30 (21%) | Exon 7 = 49 (74%)  Exon 10 = 17 (26%) |
| 18590527  Lindgren, 2008 | Microarray and sequencing | 7, 10, 13, 15 | pTa = 20, pT1 = 17, pT2-4 = 11  Gr. 1 = 5, Gr. 2 =23, Gr. 3 = 20 | **15/48 (31%)**  pTa = 9 (45%)  pT1 = 5 (29%)  pT2-4 = 1 (9%)  Gr. 1 = 4 (80%)  Gr. 2 = 8 (35%)  Gr. 3 = 3 (15%) | Exon 7 = 100% |
| 18231634  Junker, 2008 | SNaPshot assay |  | pTa = 49, pT1 = 27, pT2-4 = 14  Gr. 1 = 36, Gr. 2 = 30, Gr. 3 = 22 | **45/92 (49%)**  pTa = 34 (69%)  pT1 = 10 (37%)  pT2-4 = 1 (7%)  Gr. 1 = 26 (72%)  Gr. 2 = 18 (60%)  Gr. 3 = 1 (5%) | Exon 7 = 28 (62%)  Exon 10 = 14 (31%)  Exon 15 = 3 (7%) |
| 17803960  Miyake, 2007 | Sequencing | 7, 10, 15 | pTa = 9, pT1 = 4  Gr. 1 = 5, Gr. 2 = 7, Gr. 3 = 1 | **12/13 (92%)**  pTa =8 (89%)  pT1 = 4 (100%)  Gr. 1 = 5 (100%)  Gr. 2 = 6 (86%)  Gr. 3 = 1 (100%) | Exon 7 = 8 (62%)  Exon 10 = 5 (38%)  Exon 15 = 0 (0%) |
| 16877735  Hernandez, 2006 | Sequencing | 7, 10 | LMP = 43, pTaG1 = 251, pTaG2 = 239, pTaG3 = 88, pT1G2 = 24, pT1G3 = 119 | **385/772 (50%)**  LMP = 33 (77%)  pTaG1 = 158 (62%)  pTaG2 = 139 (58%)  pTaG3 = 30 (34%)  pT1G2 = 7 (27%)  pT1G3 = 20 (17%) | Exon 7 = 245 (62%)  Exon 10 = 152 (38%) |
| 16570285  van Oers, 2006 | SnaPshot assay |  | Hyperplasia = 30, pTaLowGr. = 4, pTaHighGr. = 1, pT1HighGr. = 3, pT2HighGr. = 1 | **9/39 (23%)**  Hyperplasia = 7 (23%)  PTaLowGr. = 2 (50%) | - NR - |
| 17668422  Tomlinson, 2007 | Sequencing | 7, 10, 15 | pTa = 71, pT1 = 29, pT2-4 = 47, pTx = 8, pTis = 3  Gr. 1 = 13, Gr. 2 = 60, Gr. 3 = 85 | **92/158 (58%)**  pTa = 24 (34%)  pT1 = 18 (62%)  pT2-4 = 40 (85%)  pTx = 7 (88%)  pTis = 3 (100%)  Gr. 1 = 5 (39%)  Gr. 2 = 20 (33%)  Gr. 3 = 67 (79%) | Exon 7 = 42 (59%)  Exon 10 = 25 (35%)  Exon 15 = 4 (6%) |
| 11745189  Kimura, 2001 | SSCP and sequencing | 7, 10, 15, 19 | pTa = 10, pT1 = 39, pT2-4 = 32  Gr. 1 = 4, Gr. 2 = 31, Gr. 3 = 46 | **25/81 (31%)**  pTa-1 = 21 (43%)  pT2-4 = 4 (13%)  Gr. 1 = 3 (75%)  Gr. 2 = 15 (48%)  Gr. 3 = 7 (15%) | Exon 7 = 13 (52%)  Exon 10 = 10 (40%)  Exon 15 = 2 (8%)  Exon 19 = 0 (0%) |
| 19621447  Otto, 2009 | SnaPshot assay | 7, 10, 15 | pTa = 31, pT1 = 3, pT2-4 = 3, pTis = 1  Gr. 1 = 20, Gr. 2 = 13, Gr. 3 = 4 | **38/38 (100%)**  **All patients harboured activating *FGFR3* mutations** | Exon 7 = 26 (67%)  Exon 10 = 12 (31%)  Exon 15 = 1(2%) |
| 19637316  Zieger, 2009 | Microarray | 7, 10 | pTa = 118, pT1 = 100, pTis = 84  Gr. 1 = 79, Gr. 2 = 15, Gr. 3 = 124 | **101/302 (33%)**  pTa = 65 (55%)  pT1 = 22 (22%)  pTis = 14 (17%)  Gr. 1 = 55 (70%)  Gr. 2 = 4 (27%)  Gr. 3 = 28 (23%) | - NR - |
