# Supplementary material for: A Meta-Analysis of the Relationship between FGFR3 and TP53 Mutations in Bladder Cancer
Source: PLoS One. 2012 Dec 13;7(12):e48993. doi: 10.1371/journal.pone.0048993 (PMC3521761; doi:10.1371/journal.pone.0048993)
Supplement: Table S2 — Overview of TP53 mutations studies in bladder carcinoma. (DOC) [file pone.0048993.s002.doc]

**Supplementary Table 2: Overview of *TP53* mutations studies in bladder carcinoma**

| Ref. PMID | Techniques | Exon | Pathological stages and grades | Frequencies of mutations (%) | Frequencies of mutations by exon (%) |
| --- | --- | --- | --- | --- | --- |
| 14577491  Babjuk, 2002 | SSCP and Sequencing | 5-8 | pTa =39, pT1 = 21  Gr. 1 = 20, Gr. 2 = 35, Gr. 3 = 5 | **1/60 (1.7%)**  One case in pTaG2 | Mutation in exon 5 |
| 16061860  Hernandez, 2005 | Sequencing | 4-9 | 119 pT1G3 | **78/119 (65.5%)** | Exon 4: 16 (20.5%)  Exon 5: 28 (35.9%)  Exon 6: 8 (10.3%)  Exon 7  13 (16.7%)  Exon 8: 24 (30.8%)  Exon 9: 7 (9.0%) |
| 16278391  Zieger, 2005 | Sequencing | 5-8 | pTaG1-2 = 19  pT1G3 or CIS = 17  Recurrent (Rc) pT1G3 or CIS = 19  pT≥2 = 36 | **15/91 (16.5%)**  pTaG1-2 = 0/19 (0%)  pT1G3/CIS = 1/17 (5.9%)  Rc pT1G3/CIS = 2/19 (10.5%)  pT≥2 = 12/41 (29.3%) | - NR - |
| 15538112  Erill, 2004 | SSCP and Sequencing | 4-8 | pTa = 36, pT1 = 23, pT≥2 = 16, pTis = 1  Gr. 1 = 10, Gr. 2 = 36, Gr. 3 = 30 | **22/76 (28.9%)** | Exon 4: 2 (8.7%)  Exon 5: 6 (26.1%)  Exon 6: 0 (0.0%)  Exon 7 : 6 (26.1%)  Exon 8: 9 (39.1%)  *(23 mutations in 22 patients)* |
| 11801555  Lu, 2002 | SSCP and Sequencing | 2-11 | pT ≤3a = 40, pT≥3b = 100  Gr.1-2 = 32, Gr. 3 = 108 | **76/140 (54.3%)** | - NR - |
| 16532037  Lindgren, 2006 | SSCP and Sequencing | 4-9 | TaG1 = 25, TaG2 = 29, TaG3 = 3,  T1G2 = 10, T1G3 = 8 | **9/75 (12%)**  TaG1 = 2/25 (8%)  TaG2 = 2/29 (7%)  TaG3 = 1/3 (33%)  T1G2 = 2/10 (20%)  T1G3 = 2/8 (25%) | - NR - |
| 14678961  Bakkar, 2003 | DHPLC and sequencing | 2-11 | pTa = 31, CIS = 1, pT1 = 30, pT2-4 = 19  Gr. 1 = 10, Gr. 2 = 29, Gr. 3 = 42 | **17/81 (21%)**  pTa = 1/31 (3%)  pT1 = 7/30 (23%)  pT2-4 = 9/19 (47%) | Exon 4: 1 (6%)  Exon 5: 5 (29%)  Exon 6: 3 (18%)  Exon 7: 5 (29%)  Exon 8: 2 (12%)  Exon 9: 1 (6%) |
| 11223675  Friedrich, 2001 | TGGE and sequencing | 5-8 | pTa = 18, pT1 = 22  Gr. 1 = 7, Gr. 2 = 28, Gr. 3 = 5 | **10/40 (25%)**  pTa = 2/18 (11.1%)  pT1 = 8/22 (36.4%)  Gr. 1 = 1/7 (14.3%)  Gr. 2 = 8/28 (28.6%)  Gr. 3 = 1/5 (20%) | Exon 5: 2 (5%)  Exon 6: 1 (2,5%)  Exon 7: 3 (7,5%)  Exon 8: 4 (10%) |
| 14534739  Dahse, 2003 | Direct sequencing | 5-9 | Primary tumors:  pTa = 10, pT1 = 8, pT2 = 2, CIS = 1  Gr. 1 = 6, Gr. 2 = 7, Gr. 3 = 7, LMP = 1  Recurrences:  pTa = 2, pT1 = 16, pT2-4 = 22  Gr. 1 = 2, Gr. 2 = 30, Gr. 3 = 8 | **26/61 (43%)**  pTa = 10/12 (83%)  pT1 = 10/24 (42%)  pT2-4= 6/22 (27%)  Gr. 1 = 5/8 (62.5%)  Gr. 2 = 16/37 (43%)  Gr. 3 = 5/15 (33%) | Exon 5: 0 (0%)  Exon 6: 16 (62%)  Exon 7: 4 (15%)  Exon 8: 6 (23%)  Exon 9: 0 (0%) |
| 18048815  George, 2007 | GeneChip | 2-8 | pTa-2N0 = 72, pT3-4N0 = 40, N+ = 38 | **55/150 (37%)**  pTa-2N0 = 19/72 (26%)  pT3-4N0 = 17/40 (42%)  N+ = 19/38 (50%) | Exon 2: 1 (2%)  Exon 3: 0 (0%)  Exon 4: 2 (4%)  Exon 5: 18 (32%)  Exon 6: 2 (4%)  Exon 7: 6 (11%)  Exon 8: 14 (25%)  Multiple mut. exon 5: 4 (7%)  Multiple mut. exon 8: 1 (2%)  Multiple mut. exon 5+8 : 7 (13%) |
| 16624482  Yurakh, 2006 | SSCP and Sequencing | 5-8 | pTa = 8, pT1 = 55, pT2-4 = 21  Gr. 1 = 15, Gr. 2 = 43, Gr. 3 = 26 | **9/84 (10.7%)** | - NR - |
| 15499621  Ryk, 2005 | SSCP and Sequencing | 5-8 | pTa = 159, pT1 = 46, pT2-3 = 74, pTis = 6 | **44/327 (13.4%)**  pTa = 14/159 (8.8%)  pT1 = 10/46 (22.2%)  pT2-4 = 18/74 (25.7%)  pTis = 0/6 (0%) | Exon 5: 12 (25%)  Exon 6: 5 (10%)  Exon 7: 13 (27%)  Exon 8: 18 (38%) |
| 12919957  Moore, 2003 | SSCP and sequencing | 5-8 | pTa = 43, pT1 = 60, pT2-4 = 44  Gr. 1 = 37, Gr. 2 = 76, Gr. 3 = 34  Patients with arsenic exposure | **45/126 (36%)**  pTa = 8/35 (23%)  pT1 = 17/51 (33%)  pT2-4 = 20/40 (50%)  Gr. 1 = 4/28 (14%)  Gr. 2 = 24/65 (37%)  Gr. 3 = 17/33 (52%) | - NR - |
| 11391594  Prescott, 2001 | DGGE and sequencing | 4-8 | pTa = 13, pT1 = 9, pT2-4 = 19, pTis = 6, pTx = 2  Low Gr. = 14, High Gr. = 35 | **19/49 (39%)**  pTa = 3/13 (23%)  pT1 = 4/9 (44%)  pT2-4 = 7/19 (37%)  pTis = 4/6 (67%)  pTx = ½ (50%)  Low Gr. = 0/14 (0%)  High Gr. = 19/35 (54%) | Exon 4 = 1 (2%)  Exon 5 = 5 (14%)  Exon 6 = 5 (14%)  Exon 7 = 13 (35%)  Exon 8 = 13 (35%) |
| 10607740  LaRue, 2000 | SSCP and sequencing | 5-8 | pTa = 21, pT1 = 29, pTx = 1  Gr. 1 = 2, Gr. 2 = 13, Gr. 3 = 36 | **16/51 (31%)**  pTa = 5/21 (24%)  pT1 = 11/29 (38%)  Gr. 1 = 0/2 (0%)  Gr. 2 = 1/13 (8%)  Gr. 3 = 15/36 (42%) | Exon 5 = 5 (29%)  Exon 6 = 2 (12%)  Exon 7 = 2 (12%)  Exon 8 = 8 (47%) |
| 9649138  Abdel-Fattah, 1998 | SSCP and sequencing | 4-9 | pTa = 9, pT1 = 10, pT2-4 = 35  Gr. 1 = 1, Gr. 2 = 20, Gr. 3 = 33 | **18/54 (33%)**  pTa = 1/9 (11%)  pT1 = 3/10 (30%)  pT2-4 = 14/35 (40%)  Gr. 1 = 0 (0%)  Gr. 2 = 4/20 (20%)  Gr. 3 = 14/33 (42%) | Exon 4 = 0 (0%)  Exon 5 = 1 (5%)  Exon 6 = 3 (16%)  Exon 7 = 8 (42%)  Exon 8 = 7 (37%)  Exon 9 = 0 (0%) |
| 9635831,  Sorlie, 1998 | CDGE | 4-9 | pT = NR  Gr 1. = 5, Gr. 2 = 9, Gr. 3 = 7  Patients with exposure to aromatic amines | **8/21 (38%)**  Gr. 1 = 0/5 (0%)  Gr. 2 = 3/9 (33%)  Gr. 3 = 5/7 (71%) | Exon 4 = 1 (11%)  Exon 5 = 1 (11%)  Exon 6 = 1 (11%)  Exon 7 = 2 (22%)  Exon 8 = 4 (45%)  Exon 9 = 0 (0%) |
| 9466649  Martone, 1998 | GC-NICI-MS and sequencing | 4-8 | - NR- | **9/45 (20%)** | Exon 4 = 0 (0%)  Exon 5 = 3 (30%)  Exon 6 = 1 (10%)  Exon 7 = 3 (30%)  Exon 8 = 3 (30%) |
| 9264274  Xu, 1997 | DGGE and sequencing | 4-9 | - NR- | **13/28 (46%)** | Exon 4 = 2 (14%)  Exon 5 = 2 (14%)  Exon 6 = 1 (8%)  Exon 7 = 2 (14%)  Exon 8 = 7 (50%) |
| 15308588  Schlichtholz, 2004 | SSCP and sequencing | 4-8 | pT a-1 = 9, pT2-4 = 21  Gr. 1 = 2, Gr. 2 = 9, Gr. 3 = 19  Patients with arylamine exposure were excluded from this analysis | **16/30 (53%)**  pTa-1 = 6/9 (67%)  pT2-4 = 10/21 (48%)  Gr. 1 = 0/2 (0%)  Gr. 2 = 3/9 (33%)  Gr. 3 = 13/19 (68%) | Exon 4 = 3 (16%)  Exon 5 = 5 (26%)  Exon 6 = 2 (11%)  Exon 7 = 1 (5%)  Exon 8 = 8 (42%) |
| 8080737  Vet, 1994 | SSCP and sequencing | 5-8 | pTa = 15, pT1 = 8, pT2-4 = 24  Gr. 1 = 12, Gr. 2 = 6, Gr. 3 = 33 | **8/47 (17%)**  pTa = 0/15 (0%)  pT1 = 0/8 (0%)  pT2-4 = 8/24 (33%) | Exon 5 = 3 (37%)  Exon 6 = 1 (13%)  Exon 7 = 1 (13%)  Exon 8 = 3 (37%) |
| 2024123  Sidransky, 1991 | DGGE and sequencing | 5-9 | pT1 = 3, pT2 = 2, pT3 = 12, pT4 = 1 | **11/18 (61%)**  pT1 = 1/3 (33%)  pT2 = 0/2 (0%)  pT3 = 10/12 (83%)  pT4 = 0/1 (0%) | Exon 4 = 1 (8%)  Exon 5 = 2 (17%)  Exon 6 = 0 (0%)  Exon 7 = 3 (25%)  Exon 8 = 6 (50%) |
| 18590527  Lindgren, 2008 | Microarray and sequencing | 4-9 | pTa = 20, pT1 = 17, pT2-4 = 11  Gr. 1 = 5, Gr. 2 =23, Gr. 3 = 20 | **11/48 (23%)**  pTa = 2/20 (10%)  pT1 = 5/17 (29%)  pT2-4 = 4/11 (36%)  Gr. 1 = 0 /5(0%)  Gr. 2 = 3 /23 (13%)  Gr. 3 = 8/30 (40%) | Exon 4 = 0 (0%)  Exon 5 = 1 (9%)  Exon 6 = 1 (9%)  Exon 7 = 5 (45%)  Exon 8 = 3 (27%)  Exon 9 = 1 (9%) |
| 15135005  Watanabe, 2004 | FASAY and sequencing | - NR - | pTa = 35, pT≥1 = 40 Gr. 1-2 = 50, Gr. 3 = 25 | **24/75 (32%)**  pTa = 5/35 (14.3%)  pT≥1 = 19/40 (47.5%) | - NR - |
| 16000567  Dekairelle, 2005 | FASAY and Sequencing | 4-8 | pTa = 37, pT1 = 9, pT≥2 = 6  Gr. 1 = 14, Gr. 2 = 11, Gr. 3 = 27 | **19/52 (36.5%)**  pTa = 8/37 (21.6%)  pT1 = 6/9 (66.7%)  pT≥2 = 5/6 (83.3%) | Exon 4: 3 (14.3%)  Exon 5: 5 (23.8%)  Exon 6: 0 (0.0%)  Exon 7: 4 (19.0%)  Exon 8: 9 (42.9%) |
| 17495352  Borkowska, 2007 | SSCP and sequencing | 5-8 | pTa = 26, pT1 = 26, pT≥2 = 29, pTis = 1  Gr. 1 = 30, Gr. 2 = 34, Gr. 3 = 18 | **16/82 (19.5%)**  pTa = 3/26 (11.5%)  pT1 = 4/26 (15.4%)  pT≥2 = 8/29 (27.6%)  pTis = 1/1 (100%) | Exon 5: 2 (6.7%)  Exon 6: 16 (53.3%)  Exon 7: 2 (6.7%)  Exon 8: 10 (33.3%) |
| 11555595  Dalbagni, 2001 | SSCP and sequencing | 5-8 | pTa = 28, pT1 = 7, pT≥2 = 6, pTis = 9  Gr. 1 = 16, Gr. 2 = 9, Gr. 3 = 16 | **23/50 (46.0%)**  pTa = 6/28 (21.4%)  pT1 = 6/7(85.7%)  pT≥2 = 4/6 (66.7%)  pTis = 7/9 (77.8%) | Exon 5: 2 (28.6%)  Exon 6: 3 (42.9%)  Exon 7: 0 (0.0%)  Exon 8: 2 (28.6%) |
| 10492244  Bernardini, 1999 | SSCP and sequencing | 5-9 | pTa = 43, pT1 = 21, pT2-4 = 40  Gr. 1 = 23, Gr. 2 = 27, Gr. 3 = 54 | **80/104 (77%)**  pTa = 40 (93%)  pT1 = 19 (90%)  pT2-4 = 21 (53%)  Gr. 1 = 22 (96%)  Gr. 2 = 24 (89%)  Gr. 3 = 34 (63%) | Exon 5 = 7 (25%)  Exon 6 = 3 (11%)  Exon 7 = 9 (32%)  Exon 8 = 7 (25%)  Exon 9 = 2 (7%) |
| 7715016  Yoshimura, 1995 | SSCP and sequencing | 4-9 | pTa = 8, pT1 =20, pTis = 2  Gr. 1 = 6, Gr. 2 = 24, Gr. 3 = 0 | **5/30 (17%)**  pTa = 0 (0%)  pT1 = 5 (25%)  pTis = 0 (0%)  Gr. 1 = 1 (17%)  Gr. 2 = 4 (17%)  Gr. 3 = 0 (0%) | Exon 4 = 0 (0%)  Exon 5 = 1 (20%)  Exon 6 = 1 (20%)  Exon 7 = 1 (20%)  Exon 8 = 2 (40%)  Exon 9 = 0 (0%) |
| 7906253  Cordon-Cardo, 1994 | SSCP and sequencing | 5-9 | pTa = 7, pT1 = 9, pT≥2 = 26  Gr. 1 = 8, Gr. 2 = 9, Gr. 3 = 25 | **14/42 (33%)**  pTa = 0 (0%)  pT1 = 2 (29%)  pT≥2 = 12 (46%)  Gr. 1 = 0 (0%)  Gr. 2 = 3 (33%)  Gr. 3 = 11 (44%) | Exon 5 = 8 (50%)  Exon 6 = 0 (0%)  Exon 7 = 3 (19%)  Exon 8 = 5 (31%)  Exon 9 = 0 (0%) |
| 7957118  Kusser, 1994 | SSCP and sequencing | 5-9 | Low Gr. = 20, High Gr. = 12 | **8/32 (25%)**  Low Gr. = 3 (15%)  High Gr. = 5 (40%) | Exon 5 = 1 (10%)  Exon 6 = 0 (0%)  Exon 7 = 3 (30%)  Exon 8 = 4 (40%)  Exon 9 = 2 (20%) |
| 18025850  Salinas-Sanchez, 2007 | SSCP and sequencing | 5-9 | pTa = 20, pT1 = 21, pT≥2 = 29, pTis = 3  Gr. 1 = 11, Gr. 2 = 22, Gr. 3 =40 | **38/73 (52%)**  pTa = 7 (35%)  pT1 = 10 (47%)  pT≥2 = 19 (65%)  pTis = 2/3 (67%)  Gr. 1 = 2 (18%)  Gr. 2 = 12 (54%)  Gr. 3 = 24 (60%) | Exon 5 = 6 (19%)  Exon 6 = 7 (22%)  Exon 7 = 3 (9%)  Exon 8 = 13 (41%)  Exon 9 = 3 (9%) |
| 10650812  Romano, 1999 | SSCP and sequencing | 5-8 | pTa = 41, pT1 = 53, pT≥2 = 12  Gr. 1 = 12, Gr. 2 = 53, Gr. 3 = 41 | **17/106 (16%)** | Exon 5 = 6 (33%)  Exon 6 = 2 (11%)  Exon 7 = 6 (33%)  Exon 8 = 4 (22%) |
| 10948316  Dahse, 2000 | SSCP and sequencing | 5-9 | pTa = 35, pT1 = 17, pT≥2 = 7, pTis = 1 | **8/66 (12%)**  **pTa = 3 (12%)**  **pT1 = 5 (8%)**  pT≥2 = 0 (0%) | Exon 5 = 3 (38%)  Exon 6 = 1 (12%)  Exon 7 = 3 (38%)  Exon 8 = 1 (12%)  Exon 9 = 0 (0%) |
