# Supplementary material for: A Meta-Analysis of the Relationship between FGFR3 and TP53 Mutations in Bladder Cancer
Source: PLoS One. 2012 Dec 13;7(12):e48993. doi: 10.1371/journal.pone.0048993 (PMC3521761; doi:10.1371/journal.pone.0048993)
Supplement: Table S3 — Overview of FGFR3 and TP53 mutations in bladder carcinoma in the two unpublished studies. (DOC) [file pone.0048993.s003.doc]

**Supplementary Table 3: Overview of *FGFR3* and *TP53* mutations in bladder carcinoma in the two unpublished studies**

| Ref: PMID first author, year | Techniques | **Pathological stages and grades** | Frequencies of mutations *TP53* / *FGFR3* |
| --- | --- | --- | --- |
| Bladder CIT Unpublished | SNaPshot followed by sequencing | **pTa = 60, pT1 = 51p, T2-4 = 103**  **G1 = 11, G2 = 52, G3 = 151** | pTaG1 = 0% / 55%  pTaG2 = 5% / 87%  pTaG3 = 11% / 33%  pT1G2 = 17% / 83%  pT1G3 = 31% / 30%  pT≥2G2 = 40% / 40%  T≥2G3 = 48% / 7% |
| Mongiat-Artus UP  unpublished | FASAY followed by allele-specific PCR | **pTa = 93, pT1 = 46, pT2-4 = 29**  **G1 = 35, G2 = 68, G3 = 62** | pTaG1 = 0% / 74%  pTaG2 = 7% / 60%  pTaG3 = 25% / 50%  pT1G1 = 0% / 50%  pT1G2 = 23% / 69%  pT1G3 = 35% / 14%  pT≥2G3 = 38% / 21% |
