# Supplementary material for: A Meta-Analysis of the Relationship between FGFR3 and TP53 Mutations in Bladder Cancer
Source: PLoS One. 2012 Dec 13;7(12):e48993. doi: 10.1371/journal.pone.0048993 (PMC3521761; doi:10.1371/journal.pone.0048993)
Supplement: Table S4 — Available individual data from unpublished, Bakkar, Lindgren, Ouerhani, and Zieger studies. (DOC) [file pone.0048993.s004.doc]

Supplementary Table 4: Available individual data from unpublished, Bakkar, Lindgren, Ouerhani, and Zieger studies

| **study** | ***TP53*** | ***FGFR3*** | **pT** | **G** |
| --- | --- | --- | --- | --- |
| Bakkar *et al.* (03) | Mut | Wild | pT1 | G2 |
| Bakkar *et al.* (03) | Wild | Wild | pT1 | G2 |
| Bakkar *et al.* (03) | Wild | Mut | pT≥2 | G3 |
| Bakkar *et al.* (03) | Wild | Wild | pT1 | G2 |
| Bakkar *et al.* (03) | Mut | Wild | pTa | G2 |
| Bakkar *et al.* (03) | Mut | Wild | pT1 | G2 |
| Bakkar *et al.* (03) | Mut | Wild | pTa | G2 |
| Bakkar *et al.* (03) | Wild | Mut | pT1 | G3 |
| Bakkar *et al.* (03) | Mut | Wild | pTa | G1 |
| Bakkar *et al.* (03) | Mut | Wild | pTa | G1 |
| Bakkar *et al.* (03) | Wild | Wild | pT1 | G3 |
| Bakkar *et al.* (03) | Wild | Wild | pT1 | G2 |
| Bakkar *et al.* (03) | Wild | Wild | pTa | G1 |
| Bakkar *et al.* (03) | Wild | Wild | pT1 | G3 |
| Bakkar *et al.* (03) | Wild | Mut | pT≥2 | G2 |
| Bakkar *et al.* (03) | Wild | Wild | pT≥2 | G3 |
| Bakkar *et al.* (03) | Wild | Wild | pT≥2 | G3 |
| Bakkar *et al.* (03) | Wild | Mut | pT≥2 | G3 |
| Bakkar *et al.* (03) | Wild | Mut | pT≥2 | G3 |
| Bakkar *et al.* (03) | Wild | Wild | pT≥2 | G3 |
| Bakkar *et al.* (03) | Wild | Wild | pT1 | G3 |
| Bakkar *et al.* (03) | Mut | Wild | pT1 | G2 |
| Bakkar *et al.* (03) | Mut | Wild | pT1 | G2 |
| Bakkar *et al.* (03) | Mut | Wild | pTa | G1 |
| Bakkar *et al.* (03) | Mut | Wild | pT1 | G2 |
| Bakkar *et al.* (03) | Mut | Mut | pTa | G2 |
| Bakkar *et al.* (03) | Wild | Wild | pTa | G2 |
| Bakkar *et al.* (03) | Mut | Wild | pT1 | G2 |
| Bakkar *et al.* (03) | Wild | Mut | pT1 | G2 |
| Bakkar *et al.* (03) | Wild | Wild | pTa | G2 |
| Bakkar *et al.* (03) | Mut | Wild | pTa | G3 |
| Bakkar *et al.* (03) | Wild | Wild | pTa | G2 |
| Bakkar *et al.* (03) | Mut | Wild | pT1 | G3 |
| Bakkar *et al.* (03) | Wild | Mut | pT≥2 | G3 |
| Bakkar *et al.* (03) | Mut | Wild | pT1 | G3 |
| Bakkar *et al.* (03) | Wild | Wild | pT1 | G3 |
| Bakkar *et al.* (03) | Mut | Wild | pTa | G2 |
| Bakkar *et al.* (03) | Wild | Wild | pT≥2 | G3 |
| Bakkar *et al.* (03) | Wild | Wild | pT1 | G2 |
| Bakkar *et al.* (03) | Wild | Mut | pT1 | G3 |
| Bakkar *et al.* (03) | Wild | Wild | pT1 | G2 |
| Bakkar *et al.* (03) | Wild | Wild | pT≥2 | G3 |
| Bakkar *et al.* (03) | Wild | Wild | pTa | G2 |
| Bakkar *et al.* (03) | Mut | Wild | pTa | G3 |
| Bakkar *et al.* (03) | Wild | Mut | pT≥2 | G3 |
| Bakkar *et al.* (03) | Wild | Wild | pT≥2 | G3 |
| Bakkar *et al.* (03) | Wild | Wild | Tis | G3 |
| Bakkar *et al.* (03) | Wild | Wild | pT≥2 | G3 |
| Bakkar *et al.* (03) | Mut | Wild | pTa | G3 |
| Bakkar *et al.* (03) | Wild | Wild | pT≥2 | G3 |
| Bakkar *et al.* (03) | Wild | Wild | pT1 | G3 |
| Bakkar *et al.* (03) | Wild | Wild | pT≥2 | G3 |
| Bakkar *et al.* (03) | Wild | Wild | pT≥2 | G3 |
| Bakkar *et al.* (03) | Mut | Wild | pTa | G3 |
| Bakkar *et al.* (03) | Wild | Wild | pT1 | G3 |
| Bakkar *et al.* (03) | Mut | Wild | pTa | G2 |
| Bakkar *et al.* (03) | Mut | Mut | pT1 | G3 |
| Bakkar *et al.* (03) | Wild | Mut | pT≥2 | G3 |
| Bakkar *et al.* (03) | Mut | Wild | pTa | G2 |
| Bakkar *et al.* (03) | Mut | Wild | pTa | G3 |
| Bakkar *et al.* (03) | Wild | Wild | pTa | G2 |
| Bakkar *et al.* (03) | Wild | Mut | pT≥2 | G3 |
| Bakkar *et al.* (03) | Mut | Wild | pTa | G2 |
| Bakkar *et al.* (03) | Mut | Mut | pT≥2 | G3 |
| Bakkar *et al.* (03) | Mut | Wild | pTa | G2 |
| Bakkar *et al.* (03) | Mut | Wild | pTa | G3 |
| Bakkar *et al.* (03) | Wild | Wild | pT1 | G2 |
| Bakkar *et al.* (03) | Wild | Wild | pTa | G3 |
| Bakkar *et al.* (03) | Wild | Mut | pT1 | G3 |
| Bakkar *et al.* (03) | Wild | Mut | pT1 | G3 |
| Bakkar *et al.* (03) | Mut | Wild | pTa | G1 |
| Bakkar *et al.* (03) | Mut | Wild | pTa | G1 |
| Bakkar *et al.* (03) | Wild | Wild | pT1 | G3 |
| Bakkar *et al.* (03) | Wild | Wild | pTa | G1 |
| Bakkar *et al.* (03) | Wild | Wild | pT1 | G2 |
| Bakkar *et al.* (03) | Wild | Wild | pTa | G1 |
| Bakkar *et al.* (03) | Mut | Wild | pTa | G1 |
| Bakkar *et al.* (03) | Mut | Wild | pTa | G1 |
| Bakkar *et al.* (03) | Wild | Mut | pT1 | G3 |
| Bakkar *et al.* (03) | Wild | Wild | pT1 | G3 |
| Bakkar *et al.* (03) | Mut | Wild | pTa | G2 |
| CIT UP | Wild | Wild | pT≥2 | G3 |
| CIT UP | Mut | Wild | pT≥2 | G3 |
| CIT UP | Wild | Wild | pT1 | G3 |
| CIT UP | Mut | Wild | pT≥2 | G3 |
| CIT UP | Mut | Wild | pT1 | G3 |
| CIT UP | Wild | Wild | pT≥2 | G3 |
| CIT UP | Wild | Wild | pT1 | G3 |
| CIT UP | Mut | Wild | pT≥2 | G3 |
| CIT UP | Wild | Wild | pT1 | G3 |
| CIT UP | Wild | Wild | pT≥2 | G3 |
| CIT UP | Mut | Wild | pT≥2 | G3 |
| CIT UP | Mut | Wild | pT≥2 | G3 |
| CIT UP | Wild | Wild | pT≥2 | G3 |
| CIT UP | Wild | Wild | pT≥2 | G3 |
| CIT UP | Mut | Wild | pT≥2 | G3 |
| CIT UP | Mut | Wild | pT1 | G3 |
| CIT UP | Mut | Mut | pT≥2 | G3 |
| CIT UP | Wild | Wild | pT1 | G3 |
| CIT UP | Mut | Wild | pT1 | G3 |
| CIT UP | Mut | Wild | pT≥2 | G3 |
| CIT UP | Wild | Mut | pT1 | G3 |
| CIT UP | Wild | Wild | pT1 | G3 |
| CIT UP | Wild | Wild | pT≥2 | G3 |
| CIT UP | Mut | Wild | pT≥2 | G3 |
| CIT UP | Mut | Wild | pT≥2 | G3 |
| CIT UP | Wild | Wild | pT≥2 | G3 |
| CIT UP | Wild | Wild | pT1 | G3 |
| CIT UP | Wild | Mut | pTa | G2 |
| CIT UP | Wild | Wild | pT≥2 | G3 |
| CIT UP | Wild | Wild | pTa | G1 |
| CIT UP | Wild | Mut | pTa | G3 |
| CIT UP | Wild | Mut | pT1 | G3 |
| CIT UP | Mut | Wild | pT1 | G3 |
| CIT UP | Mut | Wild | pT1 | G3 |
| CIT UP | Wild | Wild | pT≥2 | G3 |
| CIT UP | Mut | Wild | pTa | G3 |
| CIT UP | Wild | Wild | pT≥2 | G2 |
| CIT UP | Wild | Wild | pT1 | G3 |
| CIT UP | Mut | Wild | pT≥2 | G3 |
| CIT UP | Mut | Wild | pT≥2 | G3 |
| CIT UP | Mut | Wild | pT≥2 | G3 |
| CIT UP | Wild | Mut | pTa | G2 |
| CIT UP | Wild | Wild | pT≥2 | G3 |
| CIT UP | Wild | Wild | pT≥2 | G3 |
| CIT UP | Wild | Wild | pT≥2 | G3 |
| CIT UP | Mut | Wild | pT≥2 | G3 |
| CIT UP | Wild | Wild | pT≥2 | G3 |
| CIT UP | Wild | Wild | pT≥2 | G3 |
| CIT UP | Mut | Wild | pT≥2 | G3 |
| CIT UP | Wild | Wild | pT≥2 | G3 |
| CIT UP | Wild | Wild | pT≥2 | G3 |
| CIT UP | Wild | Wild | pT≥2 | G3 |
| CIT UP |  | Wild | pT≥2 | G3 |
| CIT UP | Wild | Wild | pT≥2 | G3 |
| CIT UP | Mut | Wild | pT≥2 | G3 |
| CIT UP | Wild | Mut | pT≥2 | G2 |
| CIT UP | Wild | Wild | pT≥2 | G3 |
| CIT UP | Wild | Wild | pTa | G2 |
| CIT UP | Wild | Wild | pTa | G1 |
| CIT UP | Mut | Mut | pT≥2 | G3 |
| CIT UP | Wild | Mut | pT1 | G2 |
| CIT UP | Mut | Wild | pT≥2 | G3 |
| CIT UP | Mut | Wild | pT≥2 | G3 |
| CIT UP | Wild | Mut |  |  |
| CIT UP | Wild | Mut | pT1 | G3 |
| CIT UP | Wild | Mut | pTa | G1 |
| CIT UP | Wild | Mut | pTa | G2 |
| CIT UP | Wild | Mut | pTa | G2 |
| CIT UP | Wild | Wild | pT≥2 | G3 |
| CIT UP | Wild | Mut | pTa | G2 |
| CIT UP | Wild | Mut | pTa | G2 |
| CIT UP | Wild | Mut | pTa | G2 |
| CIT UP | Wild | Mut | pTa | G2 |
| CIT UP | Wild | Wild | pTa | G1 |
| CIT UP | Wild | Mut | pTa | G2 |
| CIT UP | Wild | Mut | pTa | G1 |
| CIT UP | Wild | Wild | pT≥2 | G3 |
| CIT UP | Wild | Mut | pTa | G2 |
| CIT UP | Mut | Wild | pT≥2 | G3 |
| CIT UP | Wild | Mut | pTa | G2 |
| CIT UP | Mut | Mut | pTa | G2 |
| CIT UP | Mut | Wild | pT1 | G3 |
| CIT UP | Wild | Wild | pTa | G2 |
| CIT UP | Wild | Mut | pTa | G2 |
| CIT UP | Wild | Wild | pTa | G3 |
| CIT UP | Wild | Mut | pTa | G2 |
| CIT UP | Wild | Mut | pT≥2 | G2 |
| CIT UP | Wild | Wild | pT≥2 | G3 |
| CIT UP | Wild | Mut | pTa | G2 |
| CIT UP | Mut | Wild | pT1 | G3 |
| CIT UP | Wild | Mut | pTa | G3 |
| CIT UP | Mut | Wild | pT≥2 | G3 |
| CIT UP | Wild | Wild | pT≥2 | G3 |
| CIT UP | Mut | Wild | pT≥2 | G3 |
| CIT UP | Mut | Wild | pT≥2 | G3 |
| CIT UP | Wild | Wild | pT≥2 | G3 |
| CIT UP | Wild | Mut | pTa | G2 |
| CIT UP | Wild | Mut | pT1 | G3 |
| CIT UP | Mut | Wild | pT≥2 | G3 |
| CIT UP | Mut | Wild | pT≥2 | G3 |
| CIT UP | Mut | Wild | pT≥2 | G2 |
| CIT UP | Mut | Wild | pT≥2 | G2 |
| CIT UP | Wild | Wild | pT≥2 | G3 |
| CIT UP | Wild | Mut | pTa | G1 |
| CIT UP | Wild | Wild | pT≥2 | G3 |
| CIT UP | Wild | Mut | pTa | G2 |
| CIT UP | Wild | Wild | pT1 | G3 |
| CIT UP | Wild | Wild | pT1 | G2 |
| CIT UP | Mut | Wild | pT1 | G3 |
| CIT UP | Mut | Mut | pT1 | G3 |
| CIT UP | Mut | Wild | pT≥2 | G3 |
| CIT UP | Wild | Wild | pT1 | G3 |
| CIT UP | Wild | Wild | pT≥2 | G3 |
| CIT UP | Wild | Wild | pT1 | G3 |
| CIT UP | Wild | Wild | pT1 | G3 |
| CIT UP | Wild | Wild | pT1 | G3 |
| CIT UP |  | Wild |  |  |
| CIT UP | Wild | Mut | pTa | G2 |
| CIT UP | Wild | Wild | pT1 | G3 |
| CIT UP | Wild | Wild | pTa | G3 |
| CIT UP | Wild | Wild | pT1 | G3 |
| CIT UP | Mut | Wild | pT≥2 | G3 |
| CIT UP | Wild | Wild | pT≥2 | G3 |
| CIT UP | Wild | Mut | pT1 | G3 |
| CIT UP | Wild | Mut | pT1 | G2 |
| CIT UP | Wild | Wild | pT1 | G3 |
| CIT UP | Wild | Wild | pTa | G3 |
| CIT UP | Wild | Mut | pTa | G2 |
| CIT UP | Mut | Mut | pT1 | G3 |
| CIT UP | Wild | Wild | pTa | G3 |
| CIT UP | Wild | Mut | pTa | G3 |
| CIT UP | Wild | Wild | pT≥2 | G3 |
| CIT UP | Wild | Mut | pTa | G2 |
| CIT UP | Wild | Mut | pTa | G2 |
| CIT UP | Wild | Mut | pTa | G1 |
| CIT UP | Wild | Mut | pTa | G2 |
| CIT UP | Wild | Mut | pT1 | G3 |
| CIT UP | Wild | Wild | pT≥2 | G3 |
| CIT UP |  | Mut | pT1 | G3 |
| CIT UP | Wild | Wild | pT≥2 | G3 |
| CIT UP | Wild | Mut | pTa | G2 |
| CIT UP | Wild | Wild | pT≥2 | G3 |
| CIT UP | Wild | Mut | pTa | G2 |
| CIT UP | Wild | Wild | pTa | G1 |
| CIT UP |  | Mut | pTa | G2 |
| CIT UP | Mut | Wild | pT≥2 | G3 |
| CIT UP | Wild | Wild | pTa | G2 |
| CIT UP | Mut | Wild | pT≥2 | G3 |
| CIT UP | Wild | Wild | pT≥2 | G3 |
| CIT UP | Wild | Wild | pTa | G2 |
| CIT UP | Wild | Mut | pTa | G2 |
| CIT UP | Wild | Mut | pTa | G2 |
| CIT UP | Wild | Mut | pTa | G2 |
| CIT UP | Wild | Mut | pTa | G1 |
| CIT UP | Wild | Mut | pTa | G2 |
| CIT UP | Mut | Wild | pT≥2 | G3 |
| CIT UP | Wild | Wild | pTa | G1 |
| CIT UP | Wild | Mut | pTa | G1 |
| CIT UP | Wild | Mut | pTa | G2 |
| CIT UP | Mut | Wild | pT≥2 | G3 |
| CIT UP | Wild | Wild | pT≥2 | G3 |
| CIT UP | Mut | Wild | pT≥2 | G3 |
| CIT UP | Mut | Mut | pT≥2 | G3 |
| CIT UP | Mut | Wild | pT≥2 | G3 |
| CIT UP | Wild | Wild | pT≥2 | G3 |
| CIT UP | Mut | Wild | pT≥2 | G3 |
| CIT UP | Wild | Wild | pT1 | G3 |
| CIT UP | Wild | Mut | pT1 | G3 |
| CIT UP | Mut | Wild |  |  |
| CIT UP | Wild | Wild | pT≥2 | G3 |
| CIT UP | Mut | Mut | pT1 | G3 |
| CIT UP | Wild | Mut | pTa | G2 |
| CIT UP | Wild | Mut | pT1 | G2 |
| CIT UP | Wild | Wild | pTa | G3 |
| CIT UP | Wild | Mut | pT1 | G3 |
| CIT UP | Wild | Wild | pT1 | G3 |
| CIT UP | Wild | Mut | pTa | G2 |
| CIT UP | Mut | Wild | pT1 | G3 |
| CIT UP | Mut | Mut | pT1 | G2 |
| CIT UP | Wild | Wild | pT1 | G3 |
| CIT UP | Wild | Mut | pTa | G2 |
| CIT UP | Wild | Mut | pT1 | G3 |
| CIT UP | Wild | Wild | pT≥2 | G3 |
| CIT UP | Wild | Wild | pT1 | G3 |
| CIT UP | Mut | Mut | pT≥2 | G3 |
| CIT UP | Wild | Mut | pT≥2 | G2 |
| CIT UP | Wild | Wild | pT≥2 | G3 |
| CIT UP | Wild | Wild | pT≥2 | G3 |
| CIT UP | Mut | Wild | pT≥2 | G3 |
| CIT UP | Mut | Wild | pT≥2 | G3 |
| CIT UP | Mut | Wild | pT≥2 | G3 |
| CIT UP | Mut | Wild | pT1 | G3 |
| CIT UP | Wild | Wild | pT≥2 | G3 |
| CIT UP | Wild | Wild | pT≥2 | G3 |
| CIT UP | Wild | Wild | pT≥2 | G3 |
| CIT UP | Mut | Wild | pT≥2 | G3 |
| CIT UP | Wild | Wild | pT≥2 | G3 |
| CIT UP | Mut | Wild | pT≥2 | G3 |
| CIT UP | Mut | Wild | pT≥2 | G3 |
| CIT UP | Mut | Wild | pT≥2 | G3 |
| CIT UP | Mut | Wild | pT≥2 | G3 |
| CIT UP | Wild | Wild | pT≥2 | G3 |
| CIT UP | Mut | Wild | pT≥2 | G3 |
| CIT UP | Wild | Wild | pTa | G2 |
| CIT UP | Wild | Mut | pTa | G2 |
| CIT UP | Mut | Wild | pT1 | G3 |
| CIT UP | Wild | Wild | pT≥2 | G3 |
| CIT UP | Wild | Wild | pT1 | G3 |
| CIT UP | Wild | Wild | pT≥2 | G3 |
| CIT UP | Wild | Mut | pT1 | G2 |
| CIT UP | Mut | Wild | pT≥2 | G3 |
| CIT UP | Mut | Mut | pTa | G2 |
| CIT UP | Mut | Wild | pT≥2 | G3 |
| CIT UP | Mut | Mut | pT≥2 | G3 |
| CIT UP | Wild | Wild | pT1 | G3 |
| CIT UP | Wild | Mut | pT≥2 | G3 |
| CIT UP | Wild | Wild | pT≥2 | G3 |
| Lindgren *et al.* (06) | Wild | Mut | pTa | G1 |
| Lindgren *et al.* (06) | Wild | Mut | pTa | G1 |
| Lindgren *et al.* (06) | Wild | Mut | pTa | G1 |
| Lindgren *et al.* (06) | Wild | Mut | pTa | G1 |
| Lindgren *et al.* (06) | Wild | Mut | pTa | G1 |
| Lindgren *et al.* (06) | Wild | Mut | pTa | G1 |
| Lindgren *et al.* (06) | Wild | Mut | pTa | G2 |
| Lindgren *et al.* (06) | Wild | Mut | pTa | G1 |
| Lindgren *et al.* (06) | Wild | Mut | pTa | G2 |
| Lindgren *et al.* (06) | Wild | Mut | pTa | G1 |
| Lindgren *et al.* (06) | Wild | Mut | pT1 | G2 |
| Lindgren *et al.* (06) | Wild | Mut | pTa | G2 |
| Lindgren *et al.* (06) | Wild | Wild | pTa | G1 |
| Lindgren *et al.* (06) | Wild | Mut | pTa | G1 |
| Lindgren *et al.* (06) | Wild | Mut | pTa | G1 |
| Lindgren *et al.* (06) | Wild | Wild | pTa | G2 |
| Lindgren *et al.* (06) | Wild | Wild | pTa | G2 |
| Lindgren *et al.* (06) | Wild | Wild | pTa | G1 |
| Lindgren *et al.* (06) | Mut | Mut | pTa | G1 |
| Lindgren *et al.* (06) | Wild | Mut | pTa | G2 |
| Lindgren *et al.* (06) | Mut | Wild | pTa | G1 |
| Lindgren *et al.* (06) | Wild | Mut | pTa | G2 |
| Lindgren *et al.* (06) | Wild | Mut | pTa | G1 |
| Lindgren *et al.* (06) | Wild | Mut | pT1 | G2 |
| Lindgren *et al.* (06) | Wild | Wild | pT1 | G2 |
| Lindgren *et al.* (06) | Wild | Mut | pTa | G2 |
| Lindgren *et al.* (06) | Wild | Wild | pTa | G2 |
| Lindgren *et al.* (06) | Wild | Mut | pTa | G2 |
| Lindgren *et al.* (06) | Wild | Mut | pT1 | G2 |
| Lindgren *et al.* (06) | Wild | Mut | pTa | G2 |
| Lindgren *et al.* (06) | Wild | Mut | pTa | G2 |
| Lindgren *et al.* (06) | Wild | Wild | pTa | G2 |
| Lindgren *et al.* (06) | Wild | Wild | pTa | G2 |
| Lindgren *et al.* (06) | Wild | Mut | pT1 | G2 |
| Lindgren *et al.* (06) | Wild | Mut | pTa | G2 |
| Lindgren *et al.* (06) | Wild | Wild | pTa | G2 |
| Lindgren *et al.* (06) | Wild | Mut | pTa | G2 |
| Lindgren *et al.* (06) | Wild | Mut | pTa | G2 |
| Lindgren *et al.* (06) | Wild | Mut | pTa | G1 |
| Lindgren *et al.* (06) | Wild | Mut | pTa | G2 |
| Lindgren *et al.* (06) | Wild | Mut | pTa | G1 |
| Lindgren *et al.* (06) | Wild | Mut | pTa | G1 |
| Lindgren *et al.* (06) | Wild | Wild | pT1 | G3 |
| Lindgren *et al.* (06) | Wild | Wild | pTa | G2 |
| Lindgren *et al.* (06) | Wild | Wild | pT1 | G3 |
| Lindgren *et al.* (06) | Mut | Mut | pT1 | G2 |
| Lindgren *et al.* (06) | Wild | Mut | pTa | G1 |
| Lindgren *et al.* (06) | Wild | Mut | pTa | G2 |
| Lindgren *et al.* (06) | Wild | Mut | pT1 | G2 |
| Lindgren *et al.* (06) | Wild | Mut | pT1 | G2 |
| Lindgren *et al.* (06) | Wild | Mut | pTa | G1 |
| Lindgren *et al.* (06) | Wild | Wild | pTa | G3 |
| Lindgren *et al.* (06) | Wild | Wild | pTa | G2 |
| Lindgren *et al.* (06) | Mut | Wild | pTa | G2 |
| Lindgren *et al.* (06) | Wild | Mut | pTa | G2 |
| Lindgren *et al.* (06) | Wild | Mut | pT1 | G3 |
| Lindgren *et al.* (06) | Wild | Mut | pTa | G2 |
| Lindgren *et al.* (06) | Wild | Wild | pTa | G2 |
| Lindgren *et al.* (06) | Wild | Wild | pT1 | G3 |
| Lindgren *et al.* (06) | Wild | Wild | pT1 | G2 |
| Lindgren *et al.* (06) | Wild | Mut | pTa | G2 |
| Lindgren *et al.* (06) | Wild | Mut | pTa | G1 |
| Lindgren *et al.* (06) | Wild | Mut | pTa | G1 |
| Lindgren *et al.* (06) | Wild | Wild | pTa | G1 |
| Lindgren *et al.* (06) | Wild | Mut | pTa | G1 |
| Lindgren *et al.* (06) | Wild | Mut | pTa | G2 |
| Lindgren *et al.* (06) | Wild | Wild | pTa | G1 |
| Lindgren *et al.* (06) | Mut | Wild | pTa | G3 |
| Lindgren *et al.* (06) | Mut | Wild | pTa | G2 |
| Lindgren *et al.* (06) | Mut | Wild | pT1 | G3 |
| Lindgren *et al.* (06) | Mut | Wild | pT1 | G2 |
| Lindgren *et al.* (06) | Mut | Wild | pT1 | G3 |
| Lindgren *et al.* (06) | Wild | Wild | pT1 | G3 |
| Lindgren *et al.* (06) | Wild | Wild | pT1 | G3 |
| Lindgren *et al.* (06) | Wild | Wild | pTa | G3 |
| Mongiat-Artus NP | Wild | Mut | pTa | G1 |
| Mongiat-Artus NP | Mut | Mut | pTa | G2 |
| Mongiat-Artus NP | Wild | Wild | pTa | G2 |
| Mongiat-Artus NP | Wild | Mut | pTa | G3 |
| Mongiat-Artus NP | Wild | Mut | pT1 | G2 |
| Mongiat-Artus NP | Mut | Mut | pT1 | G2 |
| Mongiat-Artus NP | Mut | Wild | pT1 | G3 |
| Mongiat-Artus NP | Wild | Wild | pT1 | G3 |
| Mongiat-Artus NP | Wild | Mut | pTa | G1 |
| Mongiat-Artus NP | Wild | Wild | pTa | G2 |
| Mongiat-Artus NP | Mut | Wild | pT1 | G3 |
| Mongiat-Artus NP | Wild | Wild | pTa | G2 |
| Mongiat-Artus NP | Wild | Mut | pTa | G2 |
| Mongiat-Artus NP | Wild | Mut | pTa | G1 |
| Mongiat-Artus NP | Wild | Wild | pT≥2 | G3 |
| Mongiat-Artus NP | Mut | Mut | pT1 | G3 |
| Mongiat-Artus NP | Mut | Wild |  |  |
| Mongiat-Artus NP | Wild | Wild | pTa | G1 |
| Mongiat-Artus NP | Wild | Mut | pT≥2 | G3 |
| Mongiat-Artus NP | Wild | Wild | pT1 | G1 |
| Mongiat-Artus NP | Mut | Wild | pT1 | G2 |
| Mongiat-Artus NP | Wild | Wild | pTa | G2 |
| Mongiat-Artus NP | Mut | Wild | pT1 | G2 |
| Mongiat-Artus NP | Wild | Wild | pTa | G2 |
| Mongiat-Artus NP | Wild | Mut | pTa | G2 |
| Mongiat-Artus NP | Wild | Mut | pT1 | G1 |
| Mongiat-Artus NP | Wild | Mut | pTa | G1 |
| Mongiat-Artus NP | Wild | Mut | pT1 | G2 |
| Mongiat-Artus NP | Wild | Wild | pT≥2 | G3 |
| Mongiat-Artus NP | Wild | Mut | pTa | G1 |
| Mongiat-Artus NP | Wild | Wild | pTa | G1 |
| Mongiat-Artus NP | Wild | Wild | pT≥2 | G3 |
| Mongiat-Artus NP | Wild | Mut | pTa | G2 |
| Mongiat-Artus NP | Wild | Mut | pTa | G1 |
| Mongiat-Artus NP | Wild | Wild | pT1 | G3 |
| Mongiat-Artus NP | Mut | Mut | pTa | G2 |
| Mongiat-Artus NP | Wild | Mut | pTa | G1 |
| Mongiat-Artus NP | Mut | Wild | pT≥2 | G3 |
| Mongiat-Artus NP | Wild | Mut | pT1 | G2 |
| Mongiat-Artus NP | Wild | Wild | pT1 | G3 |
| Mongiat-Artus NP | Wild | Wild | pT1 | G3 |
| Mongiat-Artus NP | Mut | Wild | pT1 | G3 |
| Mongiat-Artus NP | Wild | Mut | pT1 | G2 |
| Mongiat-Artus NP | Wild | Wild | pTa | G2 |
| Mongiat-Artus NP | Mut | Wild | pT≥2 | G3 |
| Mongiat-Artus NP | Wild | Mut | pTa | G2 |
| Mongiat-Artus NP | Wild | Wild | pTa | G2 |
| Mongiat-Artus NP | Wild | Wild | pTa | G2 |
| Mongiat-Artus NP | Wild | Wild | pTa | G1 |
| Mongiat-Artus NP | Wild | Mut | pTa | G2 |
| Mongiat-Artus NP | Wild | Mut | pT1 | G2 |
| Mongiat-Artus NP | Wild | Wild | pTa |  |
| Mongiat-Artus NP | Wild | Wild | pT≥2 | G3 |
| Mongiat-Artus NP | Wild | Wild | pT≥2 | G3 |
| Mongiat-Artus NP | Wild | Mut | pTa | G1 |
| Mongiat-Artus NP | Wild | Mut | pTa | G1 |
| Mongiat-Artus NP | Mut | Wild | pT1 | G3 |
| Mongiat-Artus NP | Wild | Mut | pT≥2 | G3 |
| Mongiat-Artus NP | Wild | Wild | pTa | G1 |
| Mongiat-Artus NP | Wild | Wild | pT≥2 | G3 |
| Mongiat-Artus NP | Wild | Wild | pT1 | G1 |
| Mongiat-Artus NP | Wild | Mut | pT≥2 | G3 |
| Mongiat-Artus NP | Wild | Mut | pTa | G2 |
| Mongiat-Artus NP | Wild | Mut | pTa | G2 |
| Mongiat-Artus NP | Wild | Wild | pTa | G2 |
| Mongiat-Artus NP | Wild | Mut | pTa | G2 |
| Mongiat-Artus NP | Wild | Wild | pTa | G2 |
| Mongiat-Artus NP | Mut | Wild | pT≥2 | G3 |
| Mongiat-Artus NP | Wild | Mut | pTa | G2 |
| Mongiat-Artus NP | Wild | Mut | pTa | G2 |
| Mongiat-Artus NP | Wild | Mut | pT≥2 | G3 |
| Mongiat-Artus NP | Wild | Wild | pT1 | G3 |
| Mongiat-Artus NP | Wild | Mut | pTa | G1 |
| Mongiat-Artus NP | Wild | Wild | pT1 | G3 |
| Mongiat-Artus NP | Wild | Mut | pTa | G2 |
| Mongiat-Artus NP | Wild | Mut | pTa | G2 |
| Mongiat-Artus NP | Wild | Wild | pTa | G1 |
| Mongiat-Artus NP | Wild | Mut | pTa | G1 |
| Mongiat-Artus NP | Wild | Mut | pTa | G1 |
| Mongiat-Artus NP | Wild | Wild | pTa | G2 |
| Mongiat-Artus NP | Wild | Mut | pTa | G2 |
| Mongiat-Artus NP | Wild | Mut | pTa | G1 |
| Mongiat-Artus NP | Wild | Wild | pT1 | G3 |
| Mongiat-Artus NP | Wild | Wild | pT1 | G3 |
| Mongiat-Artus NP | Wild | Wild | pT≥2 | G3 |
| Mongiat-Artus NP | Mut | Wild | pT1 | G3 |
| Mongiat-Artus NP | Wild | Wild | pTa | G2 |
| Mongiat-Artus NP | Wild | Wild | pTa | G2 |
| Mongiat-Artus NP | Wild | Wild | pT≥2 | G3 |
| Mongiat-Artus NP | Wild | Wild | pT1 | G2 |
| Mongiat-Artus NP | Wild | Wild | pT≥2 | G3 |
| Mongiat-Artus NP | Mut | Wild | pT1 | G3 |
| Mongiat-Artus NP | Wild | Mut | pTa | G2 |
| Mongiat-Artus NP | Wild | Mut | pTa | G2 |
| Mongiat-Artus NP | Mut | Wild | pT≥2 | G3 |
| Mongiat-Artus NP | Mut | Wild | pT≥2 | G3 |
| Mongiat-Artus NP | Wild | Wild | pTa | G2 |
| Mongiat-Artus NP | Wild | Wild | pTa | G1 |
| Mongiat-Artus NP | Wild | Wild | pTa | G1 |
| Mongiat-Artus NP | Mut | Wild | pT1 | G3 |
| Mongiat-Artus NP | Wild | Wild | pTa | G2 |
| Mongiat-Artus NP | Mut | Mut | pTa | G2 |
| Mongiat-Artus NP | Wild | Wild | pTa | G3 |
| Mongiat-Artus NP | Wild | Mut | pTa | G2 |
| Mongiat-Artus NP | Wild | Wild | pT1 | G3 |
| Mongiat-Artus NP | Wild | Wild | pTa | G2 |
| Mongiat-Artus NP | Wild | Mut | pTa | G1 |
| Mongiat-Artus NP | Wild | Wild | pT≥2 | G3 |
| Mongiat-Artus NP | Wild | Mut | pT1 | G3 |
| Mongiat-Artus NP | Wild | Mut | pTa | G2 |
| Mongiat-Artus NP | Wild | Mut | pTa | G1 |
| Mongiat-Artus NP | Wild | Wild | pTa | G2 |
| Mongiat-Artus NP | Wild | Mut | pTa | G2 |
| Mongiat-Artus NP | Mut | Wild | pT1 | G3 |
| Mongiat-Artus NP | Wild | Wild | pTa | G2 |
| Mongiat-Artus NP | Wild | Mut | pTa | G1 |
| Mongiat-Artus NP | Wild | Mut | pTa | G2 |
| Mongiat-Artus NP | Mut | Wild | pT≥2 | G3 |
| Mongiat-Artus NP | Mut | Mut | pTa | G2 |
| Mongiat-Artus NP | Wild | Wild | pT1 | G3 |
| Mongiat-Artus NP | Mut | Mut | pT≥2 | G3 |
| Mongiat-Artus NP | Wild | Mut | pTa | G1 |
| Mongiat-Artus NP | Wild | Mut | pT1 | G3 |
| Mongiat-Artus NP | Wild | Mut | pTa | G3 |
| Mongiat-Artus NP | Wild | Wild | pT1 | G2 |
| Mongiat-Artus NP | Wild | Mut | pTa | G2 |
| Mongiat-Artus NP | Wild | Wild | pTa | G2 |
| Mongiat-Artus NP | Wild | Wild | pT1 | G3 |
| Mongiat-Artus NP | Wild | Mut | pT1 | G3 |
| Mongiat-Artus NP | Wild | Mut | pTa | G2 |
| Mongiat-Artus NP | Wild | Mut | pTa | G1 |
| Mongiat-Artus NP | Wild | Wild | pTa | G1 |
| Mongiat-Artus NP | Wild | Mut | pTa | G2 |
| Mongiat-Artus NP | Wild | Wild | pT≥2 | G3 |
| Mongiat-Artus NP | Wild | Mut | pT1 | G2 |
| Mongiat-Artus NP | Wild | Mut | pTa | G2 |
| Mongiat-Artus NP | Wild | Mut | pTa | G1 |
| Mongiat-Artus NP | Wild | Mut | pTa | G1 |
| Mongiat-Artus NP | Wild | Wild | pT1 | G3 |
| Mongiat-Artus NP | Mut | Wild | pT≥2 | G3 |
| Mongiat-Artus NP | Wild | Mut | pTa | G2 |
| Mongiat-Artus NP | Wild | Mut | pTa | G2 |
| Mongiat-Artus NP | Wild | Wild | pT1 | G3 |
| Mongiat-Artus NP | Wild | Wild |  |  |
| Mongiat-Artus NP | Wild | Wild | pTa | G2 |
| Mongiat-Artus NP | Wild | Mut | pTa | G2 |
| Mongiat-Artus NP | Wild | Wild | pT≥2 | G3 |
| Mongiat-Artus NP | Wild | Mut | pT1 | G1 |
| Mongiat-Artus NP | Wild | Mut | pT1 | G2 |
| Mongiat-Artus NP | Wild | Wild | pT1 | G3 |
| Mongiat-Artus NP | Wild | Wild | pT1 | G3 |
| Mongiat-Artus NP | Wild | Wild | pT1 | G3 |
| Mongiat-Artus NP | Wild | Wild | pTa | G2 |
| Mongiat-Artus NP | Wild | Mut | pTa | G1 |
| Mongiat-Artus NP | Wild | Mut | pTa | G1 |
| Mongiat-Artus NP | Mut | Wild | pT≥2 | G3 |
| Mongiat-Artus NP | Mut | Wild | pT≥2 | G3 |
| Mongiat-Artus NP | Wild | Mut | pT1 | G2 |
| Mongiat-Artus NP | Wild | Wild | pT≥2 | G3 |
| Mongiat-Artus NP | Wild | Mut | pT≥2 | G3 |
| Mongiat-Artus NP | Wild | Wild | pTa |  |
| Mongiat-Artus NP | Mut | Wild | pTa | G3 |
| Mongiat-Artus NP | Wild | Mut | pTa |  |
| Mongiat-Artus NP | Mut | Wild | pT≥2 | G3 |
| Mongiat-Artus NP | Mut | Wild | pT1 | G3 |
| Mongiat-Artus NP | Wild | Mut | pTa | G1 |
| Mongiat-Artus NP | Wild | Wild | pTa | G2 |
| Mongiat-Artus NP | Wild | Mut | pTa | G2 |
| Mongiat-Artus NP | Wild | Mut | pTa | G2 |
| Mongiat-Artus NP | Wild | Mut | pTa | G2 |
| Ouerhani *et al.* (09) | Wild | Wild | pT≥2 | G3 |
| Ouerhani *et al.* (09) | Mut | Wild | pT≥2 | G3 |
| Ouerhani *et al.* (09) | Mut | Wild | pT≥2 | G3 |
| Ouerhani *et al.* (09) | Mut | Wild | pT≥2 | G3 |
| Ouerhani *et al.* (09) | Wild | Mut | pT≥2 | G3 |
| Ouerhani *et al.* (09) | Mut | Wild | pT≥2 | G3 |
| Ouerhani *et al.* (09) | Wild | Mut | pT≥2 | G3 |
| Ouerhani *et al.* (09) | Wild | Wild | pT≥2 | G3 |
| Ouerhani *et al.* (09) | Mut | Wild | pT≥2 | G3 |
| Ouerhani *et al.* (09) | Mut | Wild | pT≥2 | G2 |
| Ouerhani *et al.* (09) | Mut | Wild | pT≥2 | G3 |
| Ouerhani *et al.* (09) | Wild | Mut | pT≥2 | G2 |
| Ouerhani *et al.* (09) | Mut | Wild | pT≥2 | G3 |
| Ouerhani *et al.* (09) | Wild | Mut | pT≥2 | G2 |
| Ouerhani *et al.* (09) | Mut | Mut | pT≥2 | G3 |
| Ouerhani *et al.* (09) | Wild | Wild | pT≥2 | G3 |
| Ouerhani *et al.* (09) | Mut | Wild | pT≥2 | G3 |
| Ouerhani *et al.* (09) | Mut | Wild | pT≥2 | G3 |
| Ouerhani *et al.* (09) | Mut | Mut | pT≥2 | G3 |
| Ouerhani *et al.* (09) | Mut | Mut | pT≥2 | G2 |
| Ouerhani *et al.* (09) | Mut | Wild | pT≥2 | G3 |
| Ouerhani *et al.* (09) | Mut | Wild | pT≥2 | G3 |
| Ouerhani *et al.* (09) | Wild | Wild | pT≥2 | G3 |
| Ouerhani *et al.* (09) | Wild | Wild | pT≥2 | G3 |
| Ouerhani *et al.* (09) | Wild | Wild | pT≥2 | G3 |
| Ouerhani *et al.* (09) | Mut | Wild | pT≥2 | G3 |
| Ouerhani *et al.* (09) | Mut | Wild | pT≥2 | G2 |
| Ouerhani *et al.* (09) | Mut | Wild | pT≥2 | G3 |
| Ouerhani *et al.* (09) | Mut | Wild | pT≥2 | G3 |
| Ouerhani *et al.* (09) | Mut | Wild | pT≥2 | G3 |
| Ouerhani *et al.* (09) | Wild | Wild | pT≥2 | G3 |
| Ouerhani *et al.* (09) | Wild | Wild | pT≥2 | G3 |
| Ouerhani *et al.* (09) | Wild | Wild | pT≥2 | G3 |
| Ouerhani *et al.* (09) | Mut | Wild | pT≥2 | G3 |
| Ouerhani *et al.* (09) | Wild | Wild | pT≥2 | G3 |
| Ouerhani *et al.* (09) | Mut | Wild | pT≥2 | G3 |
| Ouerhani *et al.* (09) | Mut | Wild | pT≥2 | G3 |
| Ouerhani *et al.* (09) | Wild | Wild | pT≥2 | G3 |
| Ouerhani *et al.* (09) | Mut | Wild | pT≥2 | G3 |
| Ouerhani *et al.* (09) | Mut | Wild | pT≥2 | G3 |
| Ouerhani *et al.* (09) | Wild | Wild | pT≥2 | G3 |
| Ouerhani *et al.* (09) | Wild | Wild | pT≥2 | G2 |
| Ouerhani *et al.* (09) | Mut | Wild | pT≥2 | G3 |
| Ouerhani *et al.* (09) | Wild | Wild | pT1 | G3 |
| Ouerhani *et al.* (09) | Mut | Mut | pT1 | G2 |
| Ouerhani *et al.* (09) | Wild | Mut | pT1 | G2 |
| Ouerhani *et al.* (09) | Wild | Wild | pT1 | G3 |
| Ouerhani *et al.* (09) | Mut | Wild | pT1 | G3 |
| Ouerhani *et al.* (09) | Mut | Mut | pT1 | G2 |
| Ouerhani *et al.* (09) | Wild | Wild | pT1 | G2 |
| Ouerhani *et al.* (09) | Wild | Mut | pT1 | G2 |
| Ouerhani *et al.* (09) | Mut | Wild | pT1 | G3 |
| Ouerhani *et al.* (09) | Wild | Mut | pT1 | G2 |
| Ouerhani *et al.* (09) | Wild | Wild | pT1 | G3 |
| Ouerhani *et al.* (09) | Mut | Wild | pT1 | G3 |
| Ouerhani *et al.* (09) | Mut | Wild | pTa | G3 |
| Ouerhani *et al.* (09) | Wild | Mut | pT1 | G2 |
| Ouerhani *et al.* (09) | Mut | Mut | pT1 | G2 |
| Ouerhani *et al.* (09) | Mut | Mut | pT1 | G2 |
| Ouerhani *et al.* (09) | Mut | Mut | pT1 | G3 |
| Ouerhani *et al.* (09) | Wild | Wild | pT1 | G3 |
| Ouerhani *et al.* (09) | Wild | Wild | pT1 | G2 |
| Ouerhani *et al.* (09) | Mut | Wild | pT1 | G3 |
| Ouerhani *et al.* (09) | Wild | Mut | pT1 | G3 |
| Ouerhani *et al.* (09) | Wild | Mut | pT1 | G2 |
| Ouerhani *et al.* (09) | Wild | Mut | pT1 | G2 |
| Ouerhani *et al.* (09) | Wild | Mut | pT1 | G2 |
| Ouerhani *et al.* (09) | Wild | Mut | pT1 | G3 |
| Ouerhani *et al.* (09) | Wild | Mut | pT1 | G3 |
| Ouerhani *et al.* (09) | Wild | Wild | pT1 | G3 |
| Ouerhani *et al.* (09) | Wild | Wild | pT1 | G3 |
| Ouerhani *et al.* (09) | Mut | Wild | pT1 | G3 |
| Ouerhani *et al.* (09) | Mut | Wild | pT1 | G2 |
| Ouerhani *et al.* (09) | Mut | Wild | pT1 | G3 |
| Ouerhani *et al.* (09) | Mut | Wild | pT1 | G3 |
| Ouerhani *et al.* (09) | Mut | Wild | pT1 | G3 |
| Ouerhani *et al.* (09) | Wild | Mut | pT1 | G2 |
| Ouerhani *et al.* (09) | Wild | Mut | pT1 | G2 |
| Ouerhani *et al.* (09) | Wild | Wild | pT1 | G2 |
| Ouerhani *et al.* (09) | Wild | Wild | pT1 | G2 |
| Ouerhani *et al.* (09) | Mut | Wild | pT1 | G3 |
| Ouerhani *et al.* (09) | Wild | Wild | pT1 | G3 |
| Ouerhani *et al.* (09) | Wild | Mut | pT1 | G2 |
| Ouerhani *et al.* (09) | Wild | Wild | pT1 | G2 |
| Ouerhani *et al.* (09) | Mut | Wild | pT1 | G3 |
| Ouerhani *et al.* (09) | Wild | Wild | pT1 | G3 |
| Ouerhani *et al.* (09) | Wild | Wild | pTa | G3 |
| Ouerhani *et al.* (09) | Wild | Mut | pT1 | G3 |
| Ouerhani *et al.* (09) | Mut | Mut | pT1 | G3 |
| Ouerhani *et al.* (09) | Wild | Mut | pT1 | G3 |
| Zieger *et al.* (05) |  | Wild | pTa | G2 |
| Zieger *et al.* (05) |  | Wild | pTa | G2 |
| Zieger *et al.* (05) |  | Wild | pTa | G2 |
| Zieger *et al.* (05) |  | Wild | pTa | G2 |
| Zieger *et al.* (05) |  | Mut | pTa | G2 |
| Zieger *et al.* (05) | Wild | Mut | pTa | G2 |
| Zieger *et al.* (05) | Wild | Mut | pTa | G2 |
| Zieger *et al.* (05) | Wild | Wild | pTa | G1 |
| Zieger *et al.* (05) | Wild | Mut | pTa | G2 |
| Zieger *et al.* (05) | Wild | Mut | pTa | G2 |
| Zieger *et al.* (05) | Wild | Mut | pTa | G2 |
| Zieger *et al.* (05) |  | Mut | pTa | G2 |
| Zieger *et al.* (05) | Wild | Mut | pTa | G2 |
| Zieger *et al.* (05) |  | Mut | pTa | G2 |
| Zieger *et al.* (05) | Wild | Mut | pTa | G2 |
| Zieger *et al.* (05) |  | Wild | pTa | G2 |
| Zieger *et al.* (05) | Wild | Mut | pTa | G1 |
| Zieger *et al.* (05) | Wild | Mut | pTa | G2 |
| Zieger *et al.* (05) | Wild | Mut | pTa | G2 |
| Zieger *et al.* (05) |  | Wild | pTa | G2 |
| Zieger *et al.* (05) |  | Mut | pTa | G2 |
| Zieger *et al.* (05) | Wild | Wild | pTa | G1 |
| Zieger *et al.* (05) | Wild | Mut | pTa | G2 |
| Zieger *et al.* (05) | Wild | Wild | pTa | G1 |
| Zieger *et al.* (05) | Wild | Mut | pTa | G1 |
| Zieger *et al.* (05) | Wild | Mut | pTa | G1 |
| Zieger *et al.* (05) | Wild | Mut | pTa | G2 |
| Zieger *et al.* (05) | Wild | Mut | pTa | G1 |
| Zieger *et al.* (05) | Wild | Mut | pTa | G2 |
| Zieger *et al.* (05) | Wild | Mut | pT1 | G3 |
| Zieger *et al.* (05) | Wild | Mut | pTa | G2 |
| Zieger *et al.* (05) | Wild | Mut | pTa | G2 |
| Zieger *et al.* (05) | Wild | Wild | pT1 | G3 |
| Zieger *et al.* (05) | Wild | Wild | pTa | G2 |
| Zieger *et al.* (05) |  | Wild | pTa | G3 |
| Zieger *et al.* (05) | Wild | Wild | pTa | G2 |
| Zieger *et al.* (05) | Wild | Wild | pT1 | G3 |
| Zieger *et al.* (05) | Wild | Wild | pTa | G3 |
| Zieger *et al.* (05) | Wild | Mut | pT1 | G3 |
| Zieger *et al.* (05) | Wild | Mut | pT1 | G2 |
| Zieger *et al.* (05) | Wild | Wild | pTa | G3 |
| Zieger *et al.* (05) |  | Mut | pTa | G3 |
| Zieger *et al.* (05) |  |  | pTa | G2 |
| Zieger *et al.* (05) |  | Mut | pTa | G3 |
| Zieger *et al.* (05) | Wild | Mut | pT1 | G3 |
| Zieger *et al.* (05) |  | Mut | pTa | G2 |
| Zieger *et al.* (05) | Wild | Wild | pTa | G3 |
| Zieger *et al.* (05) | Mut | Mut | pTa | G3 |
| Zieger *et al.* (05) | Mut | Mut | pTa | G2 |
| Zieger *et al.* (05) | Wild | Mut | pTa | G3 |
| Zieger *et al.* (05) | Wild | Wild | pTa | G3 |
| Zieger *et al.* (05) | Wild | Wild | pTa | G3 |
| Zieger *et al.* (05) | Wild | Wild | pTa | G3 |
| Zieger *et al.* (05) | Wild | Wild | pT1 | G3 |
| Zieger *et al.* (05) | Wild | Wild | pTa | G2 |
| Zieger *et al.* (05) | Wild | Wild | pTa | G3 |
| Zieger *et al.* (05) | Wild | Wild | pT1 | G3 |
| Zieger *et al.* (05) | Wild | Mut | pTa | G3 |
| Zieger *et al.* (05) | Wild | Mut | pTa | G3 |
| Zieger *et al.* (05) | Wild | Mut | pT1 | G3 |
| Zieger *et al.* (05) | Mut | Wild | pT1 | G3 |
| Zieger *et al.* (05) | Wild | Wild | pTa | G2 |
| Zieger *et al.* (05) | Wild | Mut | pTa | G2 |
| Zieger *et al.* (05) | Wild | Mut | pTa | G3 |
| Zieger *et al.* (05) | Wild | Mut | pT1 | G3 |
| Zieger *et al.* (05) | Wild | Mut | pTa | G2 |
| Zieger *et al.* (05) | Wild | Mut | pT1 | G3 |
| Zieger *et al.* (05) | Wild | Wild | pTa | G3 |
| Zieger *et al.* (05) | Mut | Wild | pT1 | G3 |
| Zieger *et al.* (05) | Wild | Wild | pTa | G3 |
| Zieger *et al.* (05) |  | Mut | pT1 | G3 |
| Zieger *et al.* (05) |  | Wild | pT1 | G3 |
| Zieger *et al.* (05) |  | Wild | pTa | G2 |
| Zieger *et al.* (05) | Wild | Wild | pT1 | G3 |
| Zieger *et al.* (05) | Wild | Wild | pT1 | G3 |
| Zieger *et al.* (05) | Wild | Wild | pT≥2 | G3 |
| Zieger *et al.* (05) | Wild | Wild | pT1 | G3 |
| Zieger *et al.* (05) |  | Wild | pT1 | G3 |
| Zieger *et al.* (05) | Wild | Wild | pT≥2 | G3 |
| Zieger *et al.* (05) | Wild | Wild | pTa | G3 |
| Zieger *et al.* (05) | Wild | Wild | pT≥2 | G3 |
| Zieger *et al.* (05) | Wild | Mut | pT1 | G3 |
| Zieger *et al.* (05) | Wild | Mut | pT1 | G3 |
| Zieger *et al.* (05) | Wild | Mut | pT≥2 | G3 |
| Zieger *et al.* (05) | Wild | Mut | pT1 | G3 |
| Zieger *et al.* (05) | Wild | Mut | pT≥2 | G3 |
| Zieger *et al.* (05) | Wild | Wild | pT1 | G3 |
| Zieger *et al.* (05) | Wild | Wild | pTa | G2 |
| Zieger *et al.* (05) | Mut | Wild | pT1 | G3 |
| Zieger *et al.* (05) | Mut | Wild | pT1 | G3 |
| Zieger *et al.* (05) | Mut | Wild | pT≥2 | G3 |
| Zieger *et al.* (05) | Wild | Mut | pTa | G3 |
| Zieger *et al.* (05) | Wild | Mut | pT≥2 | G3 |
| Zieger *et al.* (05) |  | Wild | pTa | G2 |
| Zieger *et al.* (05) | Mut | Wild | pT≥2 | G3 |
| Zieger *et al.* (05) | Mut | Wild | pT1 | G3 |
| Zieger *et al.* (05) | Mut | Wild | pT≥2 | G3 |
| Zieger *et al.* (05) | Wild | Wild | pT1 | G3 |
| Zieger *et al.* (05) | Wild | Wild | pT≥2 | G3 |
| Zieger *et al.* (05) | Mut | Mut | pT1 | G3 |
| Zieger *et al.* (05) | Mut | Mut | pT≥2 | G3 |
| Zieger *et al.* (05) | Wild | Mut | pTa | G2 |
| Zieger *et al.* (05) | Mut | Mut | pT1 | G3 |
| Zieger *et al.* (05) | Wild | Wild | pTa | G3 |
| Zieger *et al.* (05) |  | Wild | pT1 | G3 |
| Zieger *et al.* (05) | Mut | Wild | pT≥2 | G3 |
| Zieger *et al.* (05) | Wild | Wild | pT1 | G2 |
| Zieger *et al.* (05) | Wild | Wild | pT1 | G3 |
| Zieger *et al.* (05) |  | Wild | pT1 | G3 |
| Zieger *et al.* (05) | Wild | Wild | pT1 | G3 |
| Zieger *et al.* (05) |  | Wild | pT≥2 | G3 |
| Zieger *et al.* (05) | Mut | Mut | pT1 | G3 |
| Zieger *et al.* (05) | Wild | Wild | pT1 | G3 |
| Zieger *et al.* (05) | Mut | Wild | pTa | G3 |
| Zieger *et al.* (05) | Wild | Mut | pTa | G3 |
