# Supplementary material for: A Meta-Analysis of the Relationship between FGFR3 and TP53 Mutations in Bladder Cancer
Source: PLoS One. 2012 Dec 13;7(12):e48993. doi: 10.1371/journal.pone.0048993 (PMC3521761; doi:10.1371/journal.pone.0048993)
Supplement: Table S5 — Joint distribution of FGFR3 and P53 mutations frequencies by stage (T) and grade (G) group. (DOC) [file pone.0048993.s005.doc]

| ***FGFR3*/*TP53*** | **Wild/Wild** | **Mut/Wild** | **Mut/Mut** | **Wild/Mut** |
| --- | --- | --- | --- | --- |
| **Ta G1-2** | 8 | 15 | 1 | 0 |
| **Ta G3** | 1 | 6 | 0 | 0 |
| **T1 G2** | 7 | 6 | 0 | 1 |
| **T1 G3** | 8 | 2 | 1 | 5 |
| **T2-4 G2** | 0 | 0 | 0 | 1 |
| **T2-4 G3** | 10 | 0 | 1 | 7 |

| ***FGFR3*/*TP53*** | **Wild/Wild** | **Mut/Wild** | **Mut/Mut** | **Wild/Mut** |
| --- | --- | --- | --- | --- |
| **Ta G1-2** | 10 | 38 | 2 | 0 |
| **Ta G3** | 5 | 3 | 0 | 1 |
| **T1 G2** | 1 | 4 | 1 | 0 |
| **T1 G3** | 21 | 9 | 3 | 11 |
| **T2-4 G2** | 1 | 3 | 0 | 2 |
| **T2-4 G3** | 47 | 1 | 5 | 43 |

| ***Frequency m***issing = 3 |
| --- |

| ***FGFR3*/*TP53*** | **Wild/Wild** | **Mut/Wild** | **Mut/Mut** | **Wild/Mut** |
| --- | --- | --- | --- | --- |
| **T1 G3** | 41 | 9 | 11 | 58 |

| ***FGFR3*/*TP53*** | **Wild/Wild** | **Mut/Wild** | **Mut/Mut** | **Wild/Mut** |
| --- | --- | --- | --- | --- |
| **Ta G1-2** | 13 | 37 | 1 | 3 |
| **Ta G3** | 2 | 0 | 0 | 1 |
| **T1 G2** | 2 | 6 | 1 | 1 |
| **T1 G3** | 5 | 1 | 0 | 2 |

| ***FGFR3*/*TP53*** | **Wild/Wild** | **Mut/Wild** | **Mut/Mut** | **Wild/Mut** |
| --- | --- | --- | --- | --- |
| **Ta G1-2** | 30 | 52 | 4 |  |
| **Ta G3** | 1 | 2 |  | 1 |
| **T1 G2** | 2 | 8 | 1 | 2 |
| **T1 G3** | 16 | 3 | 1 | 9 |
| **T2-4 G3** | 13 | 5 | 1 | 10 |

| ***FGFR3*/*TP53*** | **Wild/Wild** | **Mut/Wild** | **Mut/Mut** | **Wild/Mut** |
| --- | --- | --- | --- | --- |
| **Ta G3** | 1 | 0 | 0 | 1 |
| **T1 G2** | 5 | 10 | 4 | 1 |
| **T1 G3** | 8 | 5 | 2 | 10 |
| **T2-4 G2** | 1 | 2 | 1 | 2 |
| **T2-4 G3** | 12 | 2 | 2 | 21 |

| ***FGFR3*/*TP53*** | **Wild/Wild** | **Mut/Wild** | **Mut/Mut** | **Wild/Mut** |
| --- | --- | --- | --- | --- |
| **Ta G1-2** | 8 | 20 | 0 | 0 |
| **Ta G3** | 10 | 5 | 1 | 1 |
| **T1 G2** | 1 | 1 |  |  |
| **T1 G3** | 7 | 5 | 2 | 2 |

| ***Frequency missing = 16*** |
| --- |
